# Mesenchymal-derived extracellular vesicles enhance microglia-mediated synapse remodeling after cortical injury in aging Rhesus monkeys

**DOI:** 10.1186/s12974-023-02880-0

**Published:** 2023-09-02

**Authors:** Yuxin Zhou, Hrishti Bhatt, Chromewell A. Mojica, Hongqi Xin, Monica A. Pessina, Douglas L. Rosene, Tara L. Moore, Maria Medalla

**Affiliations:** 1 Department of Anatomy & Neurobiology, Boston University, Chobanian & Avedisian School of Medicine, Boston, MA 02118 USA; 2 Department of Neurology, Henry Ford Health Systems, Detroit, MI 48202 USA; 3 Center for Systems Neuroscience, Boston University, Boston, MA 02215 USA

**Keywords:** Cortical injury, Synaptic plasticity, Neuroinflammation, Extracellular vesicles, Microglia, C1q

## Abstract

Understanding the microglial neuro-immune interactions in the primate brain is vital to developing therapeutics for cortical injury, such as stroke or traumatic brain injury. Our previous work showed that mesenchymal-derived extracellular vesicles (MSC-EVs) enhanced motor recovery in aged rhesus monkeys following injury of primary motor cortex (M1), by promoting homeostatic ramified microglia, reducing injury-related neuronal hyperexcitability, and enhancing synaptic plasticity in perilesional cortices. A focal lesion was induced via surgical ablation of pial blood vessels over lying the cortical hand representation of M1 of aged female rhesus monkeys, that received intravenous infusions of either vehicle (veh) or EVs 24 h and again 14 days post-injury. The current study used this same cohort to address how these injury- and recovery-associated changes relate to structural and molecular interactions between microglia and neuronal synapses. Using multi-labeling immunohistochemistry, high-resolution microscopy, and gene expression analysis, we quantified co-expression of synaptic markers (VGLUTs, GLURs, VGAT, GABARs), microglia markers (Iba1, P2RY12), and C1q, a complement pathway protein for microglia-mediated synapse phagocytosis, in perilesional M1 and premotor cortices (PMC). We compared this lesion cohort to age-matched non-lesion controls (ctr). Our findings revealed a lesion-related loss of excitatory synapses in perilesional areas, which was ameliorated by EV treatment. Further, we found region-dependent effects of EVs on microglia and C1q expression. In perilesional M1, EV treatment and enhanced functional recovery were associated with increased expression of C1q + hypertrophic microglia, which are thought to have a role in debris-clearance and anti-inflammatory functions. In PMC, EV treatment was associated with decreased C1q + synaptic tagging and microglia–spine contacts. Our results suggest that EV treatment may enhance synaptic plasticity via clearance of acute damage in perilesional M1, and thereby preventing chronic inflammation and excessive synaptic loss in PMC. These mechanisms may act to preserve synaptic cortical motor networks and a balanced normative M1/PMC synaptic function to support functional recovery after injury.

**Supplementary Information:**

The online version contains supplementary material available at 10.1186/s12974-023-02880-0.

## Introduction

Cortical injury causes neuronal damage and loss of synaptic connections, leading to significant cognitive and behavioral impairments [1, 2]. Brain plasticity enables functional recovery after cortical injury, which is modulated by neuro-inflammatory responses [3]. Microglia, as the immune cells of the brain, can promote neuronal plasticity and recovery via phagocytosis of damaged pre- and post-synaptic elements, and the release of neurotrophic factors to facilitate synapse turn-over [4]. On the other hand, chronic inflammatory activity of microglia can exacerbate neuronal damage and prevent recovery [5]. However, once inflammation subsides, microglia can secrete anti-inflammatory cytokines such as IL-10 and TGF-β that can promote neuronal plasticity and repair [5, 6]. Thus, post-injury recovery is dependent on the interplay and balance of inflammatory responses and facilitation of neuronal synaptic plasticity, which is key to developing effective therapeutics, but not well understood especially in the primate brain.

Recent studies from our group demonstrated that intravenous administration of extracellular vesicles (EVs) derived from bone marrow mesenchymal stromal cells (MSCs) facilitate recovery of motor function after cortical injury in the primary motor cortex (M1) in aged female rhesus monkeys [7–9]. EVs are nanovesicles containing various biomaterials including proteins, DNAs, RNAs and miRNAs, and are involved in cell-to-cell signaling [10, 11]. Intravenous administration of EVs derived from MSCs in a rat model of traumatic brain injury enhanced spatial learning and sensorimotor functional recovery, increased vascular density, angiogenesis, and neurogenesis, promoted distal axon growth and reduced brain inflammation [9, 10]. In a series of studies from our group using our aged rhesus monkey model of cortical injury that involves induced damage in the hand representation of M1, we found that intravenous infusions of MSC-EVs 24 h and again at 14 days post-injury enhanced recovery of fine motor function of the hand. Follow-up studies have shown that this EV-mediated recovery is associated with an enhanced shift from inflammatory to homeostatic microglial ramified morphologies, reducing injury-related excitotoxic neuronal hyperexcitability, and enhancing dendritic and synaptic plasticity in perilesional M1 and premotor cortex (PMC) [12–14]. However, whether there is a link between these microglial and synaptic changes associated with injury and recovery is unknown. Therefore, the current study aims to build on these previous findings by assessing the interplay between the microglial-mediated responses and changes in neuronal synaptic plasticity in tissue from these same monkeys.

Cortical injury leads to neuronal damage and increased glutamate synaptic spillover that causes acute excitotoxicity and loss of synapses and connections (for review, see [3, 15]). Early in the recovery period, microglia can function to phagocytose and clear damaged neurons and synapses. However, once debris is cleared, anti-inflammatory and trophic microglial signaling take over [16] to promote neurite re-growth, synapse remodeling [17–20] and compensate for synapse loss, hence, restoring the excitatory:inhibitory balance of circuits (for review, see [3, 18, 21–25].

One important signaling pathway implicated in this microglia-mediated synaptic plasticity is the complement system. Microglia can produce and release C1q, a protein that can tag unwanted or damaged synapses, for initiation of the complement pathway cascade. Depending on the downstream effectors, activation of the complement pathway may lead to synapse phagocytosis [26] or other processes that can promote plasticity, such as trogocytosis—the partial phagocytosis of synapses [27]. While the complement cascade plays an important role in debris clearance and plasticity after brain injury, the expression of complement proteins in microglia and tagging of neuronal synaptic structures in the primate cortex is unknown. Further, in a rodent model of cortical injury, a subset of immune-activated microglia are shown to express C1q, and are thought to have a protective role, promoting the clearance of apoptotic cells, secretion of anti-inflammatory cytokines, and suppressing production of pro-inflammatory cytokines [6, 8, 28, 29]. These pro- versus anti-inflammatory functional microglial states are associated with a continuum of morphological phenotypic shifts that are largely unexplored in the primate brain. Upon immune activation, such as after cortical injury, microglia transition from a ramified homeostatic state to a hypertrophic or amoeboid phagocytic state. However, these subpopulations are heterogenous, especially the transitional hypertrophic state. Indeed immune-activated microglia can shift towards diverse phenotypes polarized towards either pro- or anti-inflammatory functions, expressing distinct markers [30–32]. Our previous study showed that EV treatment after cortical injury was associated with greater microglial ramification [13], but the molecular and proteomic profiles of these microglia are unknown.

Here, we investigated whether EV treatment alters the expression of synaptic markers for excitatory and inhibitory transmission, and structural and molecular markers of microglia–synapse interactions after cortical injury in perilesional M1 and PMC. Specifically, we assessed C1q expression on neuronal synapses as well as on distinct microglial morphological subtypes, and further determined whether these microglia and synapse outcome measures are correlated with functional recovery. The current study presents evidence of a link between the enhancement of microglial anti-inflammatory phenotypes and dampening of lesion-related synaptic loss and phagocytosis, which are shown to be enhanced by EV treatment.

## Material and methods

### Experimental subjects and experimental design

A total of 13 aged Rhesus monkeys (*Macaca mulatta*), ranging from 16 to 26 years old (non-lesion control *n = *2 females and *n = *1 male; lesion + vehicle *n = *5 females; lesion + EV *n = *5 females), acquired from either national primate centers or private vendors were used for this study. These monkeys were the same cohort used in our previous studies [12–14]. All monkeys were prescreened to exclude animals with brain abnormality or history of neurological diseases, chronic diseases, diabetes, or malnutrition [33]. They were housed in the Animal Science Center of Boston University Medical Campus under a 12-h light/dark cycle. All experimental procedures using animals were approved by the Boston University Institutional Animal Care and Use Committee (IACUC), and performed in accordance with the Guide for the Care and Use of Laboratory Animals from the National Institutes of Health Office of Laboratory Animal Welfare. Tissue from the lesion group was obtained from the cohort of female monkeys used in our previous study [12]. All of the lesioned monkeys in this study were aged females, as these were the only monkeys we could obtain at the time of study due to animal shortage.

**Fig. 1:**
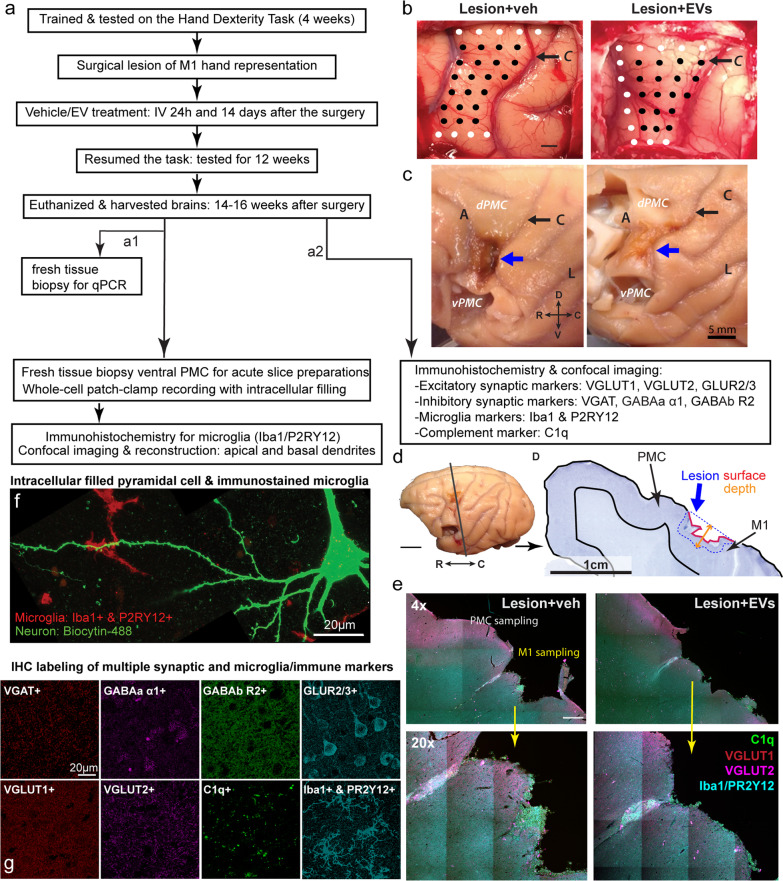
Experimental design and representative images of immunolabeled markers, lesion, and sampling location. **a** Experimental workflow as described in Moore et al., [12]. The brains were harvested 14 to 16 weeks after the surgery using two methods: (*a1*) During Krebs buffer perfusion, 1–2 cm fresh tissue block was harvested from the ventral perilesional M1 and PMC, with caudal 1/4 processed for qPCR and the rostral ¾ was cut into 300 µm acute slices for whole-cell patch-clamp recording and intracellular filling of layer 3 pyramidal cells. *(a2)* The remainder of the brain containing the lesion and dorsal PMC was fixed with 4% paraformaldehyde then cut into serial coronal sections for IHC labeling. **b**, **c** Photographs showing the hand representation (sites with black dots) mapped with electrical stimulation of M1 and (b) the lateral surface of the fixed brain (c) showing the M1 and PMC, with the lesion area (blue arrow), and locations of sampled sites in dorsal (dPMC) and ventral (vPMC taken out) PMC, as described in previous studies [12, 14]. Sulci: A, arcuate; C, central (black arrow); L, lateral; axes: D, dorsal; V, ventral; M, medial; L, lateral. **d** Photograph of ipsilesional hemisphere and coronal section (gray matter with intact pia indicated with black line) thorough the surgical lesion (lesion volume is outlined with blue dotted line, lesion surface indicated with red line and depth measurement with orange arrow), with black arrows indicating perilesional M1 underlying the lesion, and adjacent dorsal PMC. **e** Example confocal images of multi-channel IHC fluorescence labeling tiled (4 × low mag yellow inset shown in 20×) in M1 and PMC from veh or EV group. Sampling sites are shown in yellow (M1) and white (PMC). **f-g** Example maximum z-projection of high-resolution confocal images used for analyses: **f** Representative pyramidal neurons intracellularly filled with 1% biocytin and visualized with Alexa 488 streptavidin conjugate, together with immunolabeled microglia. **g** Representative images showing immunolabelling of synaptic, microglial, and complement markers that were immuno-labeled included

The experimental design and workflow are summarized in Fig. 1a. These monkeys were trained on a fine motor task, the hand dexterity task, for a total of 4 weeks and randomly assigned into the EV-treated group (*n = *5) or vehicle group (*n = *5), as described [12]. A surgical lesion was then made in the hand representation of the primary motor cortex (M1) (Fig. 1b-d). Two weeks after the surgery, monkeys began re-testing on the motor task for 12 weeks to assess the degree and nature of recovery (Fig. 1a). A separate cohort of age-matched non-lesion monkeys of both sexes (*n = *3: 2 females and 1 male), which were part of a larger study on aging, was used for non-lesion control comparisons.

### Surgical lesion of M1 hand representation and post-injury treatment

Surgical procedures to induce cortical injury were employed in the lesion cohort, as described [12, 14]. Briefly, each monkey was sedated with ketamine (10 mg/kg) and anesthetized with intravenous sodium pentobarbital (15–25 mg/kg). The head was stabilized in a stereotactic apparatus, and a midline skin incision was made and the temporalis muscle was reflected. A bone flap centered over the frontal and parietal lobes, approximately 40 mm (anterior to posterior) and 35 mm (medial to lateral), was made. The cortex spanning the premotor areas posterior to the arcuate sulcus to the primary motor cortex in the precentral gyrus, in front of the central sulcus, were exposed by incising the dura. Electrical stimulation was delivered systematically to the precentral gyrus using a small monopolar silver ball electrode placed on the surface of the pia to evoke movements in the upper extremity. Stimulation sites were 2 × 2 mm apart as shown in Fig. 1b. Monopolar stimulus pulses (duration = 250 µsec; amplitudes = 2.0 to 3.0 mA) were delivered at each site, in single pulses once every 2 s, and then in a short train of four pulses at 100 Hz. During each stimulation, a trained observer recorded muscle movements (e.g., distinct movement of muscle) in specific areas of the digits, hand, forearm or arm, both visually and by palpation. The intensity of motor responses was graded on a scale of 1 to 3 (barely visible to maximal). Specific stimulation sites with the lowest threshold and highest motor response were marked on a calibrated photograph of the exposed cortex, creating a cortical surface map of the hand area that was used to guide placement of the injury (Fig. 1b).

Using the map, cortical injury was induced by inserting a small glass suction pipette under the pia to bluntly transect the small arterioles penetrating the underlying cortex. Since the hand representation is known to extend down the rostral bank of the central sulcus, the lesion was extended into the fundus of sulcus, taking care to not damage the somatosensory areas on the caudal bank. Representative photos of the cortical map and lesion for one EV treated and one Veh monkey are shown in Figs. 1b and c [12, 14]. The monkeys were recovered for 2 weeks and then re-tested on the hand dexterity task (for 12 weeks), until tissue harvesting, as described (Fig. 1a) [12]. At 24 h and 14 days after the injury, monkeys were treated with either vehicle control or EV intravenously. The EVs were extracted from MSCs harvested from the bone marrow of a young adult monkey, as described [12, 14]. For bone marrow extraction, the monkey was sedated with ketamine (10 mg/kg) and anesthetized with intravenous sodium pentobarbital. Bone marrow extracted from the iliac crest was shipped to Henry Ford Health Systems, where the MSCs were isolated and cultured in vitro for EV collection, as described [9, 10, 12, 13]. EVs were then shipped back to Boston University for intravenous administration in monkeys (EVs were administered at 4 × 10^11^ particles). All researchers involved in the current study were blinded to the treatment groups for all procedures and experiments.

### Perfusion and brain tissue preparation

Between 14 and 16 weeks after the injury, monkeys were sedated with ketamine (10 mg/kg), anesthetized with intravenous sodium pentobarbital (15–25 mg/kg) for perfusion and brain harvesting. Monkeys were perfused using a two-stage Krebs-paraformaldehyde (PFA) perfusion method as described [14]. First, ice-cold Krebs–Henseleit buffer (6.4 mM Na_2_HPO_4_, 1.4 mM Na_2_PO_4_, 137 mM NaCl, 2.7 mM KCl, 5 mM glucose, 0.3 mM CaCl_2_, 1 mM MgCl_2_, pH7.4) was perfused to facilitate collecting fresh tissue biopsies by extracting a tissue block just ventral to the lesion, containing mainly ventral PMC (Fig. 1c). Once removed, the block was transferred to oxygenated (95% O_2_, 5% CO_2_) ice-cold Ringer’s solution (26 mM NaHCO_3_, 124 mM NaCl, 2 mM KCl, 3 mM KH_2_PO_4_, 10 mM glucose, 1.3 mM MgCl_2_, pH 7.4), as described [14]. A small piece of the caudal part of the block was trimmed and flash frozen over dry ice for qPCR. The remaining ventral PMC block was then sectioned into 300-µm coronal acute slices with a vibratome for in vitro whole-cell patch clamp recording and intracellular filling [14, 34].

Following the Krebs perfusion and tissue extraction, the rest of the brain, which included the perilesional M1 and dorsal PMC (Fig. 1d), was perfusion fixed with 4L of 4% PFA (30 °C, pH7.4) and blocked in situ in the coronal plane [13]. The brain was then removed from the skull, post-perfusion fixed overnight in 4% PFA and cryoprotected in 0.1 M phosphate buffer (PB) with 10% glycerol, and 2% DMSO, and then in buffer with 2% DMSO and 20% glycerol [13]. Brains were flash-frozen in -75 °C isopentane and stored at -80 °C before being cut on a microtome in the coronal plane, into interrupted series containing eight series of 30 µm sections and one series of 60 µm sections [13]. One series was mounted and Nissl-stained with thionin for lesion validation and reconstruction (Fig. 1d). The rest of the sections were collected in phosphate buffer with 15% glycerol and stored at -80 °C for later processing.

The extent of lesion was validated histologically in the mounted thionin-stained sections. Lesion volume was determined for each monkey, as described previously [12, 14]. Briefly, first, the calibrated photograph of the lateral surface of each brain acquired after perfusion was analyzed to determine the surface area of the lesion using the Scale and Measurement tools in FIJI/Image J (https://imagej.net/Fiji; 1997–2016; RRID:SCR_002285; Fig. 1b-d). Next, serial thionin-stained coronal sections through the lesion (Fig. 1d) were digitized using a Nikon Microscope equipped with NIS Elements software (Nikon Instruments, Inc, Melville, NY). The depth of the lesion was measured by drawing a line from the pial boundary estimated from the intact adjacent cortex to the deepest extent of gliosis (Fig. 1d, orange arrow), on 5 representative thionin stained serial sections evenly spaced throughout the lesion. Three depth measurements were obtained from each section, resulting in 15 measurements for each monkey, which were averaged. The total lesion volume per monkey was then determined by multiplying the surface area by the average depth.

### Immunohistochemistry on serial sections through the lesion

To assess pre- and post-synaptic structures together with neuro-immune markers after injury, we used multi-channel immunohistochemical (IHC) fluorescence labeling on 30 or 60-µm coronal sections through the lesioned M1 and the dorsal PMC (Fig. 1f; *n = *1–2 sections per case; Additional file 1: Table S1, S2). We labeled a total of 9 markers in 13 monkeys by running six batches of IHC experiments with distinct combinations of 3–4 of antibodies (Additional file 1: Table S1, Fig. 1f, g). To control for potential batch effects and methodological between- and within-section variability, we employed the following: (i) for each experiment, we used sections through the lesion, carefully matched in rostro-caudal level across subjects; (ii) for every batch of immunolabeling, we made sure to stain on a second section a subset of markers that are well validated and have demonstrated highly consistent staining across our previous studies (VGAT, VGLUT2 and Iba1); and (iii) we ensured that we imaged multiple fields per area at highly consistent distances from the lesion. The perilesional cortex extends about 1 mm deep to the lesion surface. Thus, we ensured that we imaged multiple perilesional fields at consistent distances away from the lesioned surface in each subject.

Synaptic structures were labeled with presynaptic vesicular glutamate (VGLUT1 and VGLUT2) and GABAergic (γ-aminobutyric acid) transporters (VGAT), as well as postsynaptic glutamate α-amino-3-hydroxy-5-methyl-4-isoxazolepropionic acid (AMPA) (GLUR2/3) and GABA (GABA_a_ α1, and GABA_b_ R2) receptor subunits. To assess the microglia activity, we immuno-labeled two microglia markers: ionized calcium binding adaptor molecule 1 (Iba1)—a pan microglial marker, and purinergic receptor P2Y, G-protein coupled, 12 (P2RY12)—a purigenic receptor associated with motility and homeostasis [35, 36]. We also immunolabeled C1q, an integral complement pathway protein that is an initiator of the classic complement pathway inflammatory cascade [26]. Previous studies in rodents suggest that upregulated C1q is associated with neuronal damage and microglial-mediated phagocytosis of synapses [37].

All the sections were incubated in 50 mM glycine for one hour and were washed with 0.01 M PBS. Antigen retrieval was performed via incubation in 10 mM citrate buffer (pH = 8.5) at 60–65 °C for 20 min. Sections were then washed with 0.01 M PBS and incubated in the pre-block solution [5% bovine serum albumin (BSA), 5% normal donkey serum (NDS), 0.2% Triton X-100 in 0.01 M PBS] for one hour at room temperature. Sections were incubated in primary antibodies in carrier solution (0.2% BSA, 1% NDS, 0.1% Triton X-100 in 0.1 M PB) at 4 °C for 72 h. We used primary antibodies to goat VGLUT1 (1: 500, Synaptic Systems, Cat# 135,307, RRID: AB_2619821), guinea pig VGLUT2 (1:1000, Synaptic Systems, Cat# 135,404, RRID: AB_887884), rabbit GLUR2/3 (1:500, Millipore Cat# 07–598, RRID:AB_11213931), guinea pig VGAT (1:400, Synaptic Systems, Cat# 131,004, RRID: AB_887873), rabbit GABA_a_ α1 (1:500, Abcam, AB33299, RRID: AB_732498), mouse GABA_b_ R2 (1:1000, LSBio, LS-C285897). For the microglia, we used a combination of rabbit P2RY12 (1:250, Novus Biologicals, NBP2-33,870) and rabbit Iba1 (1:500, Wako, Cat# 019–19741, RRID: AB_839504) to enhance the staining of processes [38]. For C1q marker, we used mouse C1q (1:400, Abcam, AB71940, RRID: AB_10711046; see Additional file 1: Table S1). Brain sections were washed with PBS and incubated for 24 h at 4 °C in donkey secondary IgG antibodies conjugated to fluorescence probes (1:200): AlexaFluor 488 donkey anti-guinea pig IgG (Jackson Immuno Research Labs, Cat# 706–545-148, RRID: AB_2340472), Alexa Fluor 546 donkey anti-goat IgG (ThermoFisher Scientific, Cat# A-11056, RRID: AB_2534103), Alexa Fluor 546 donkey anti-mouse IgG (ThermoFisher Scientific, Cat# A10036, RRID: AB_2534012) and biotinylated donkey anti-rabbit (Jackson Immuno Research Labs, Cat# 711–065-152, RRID: AB_2340593). During all primary and secondary incubations, sections were microwaved using the low-wattage PELCO Biowave (TED PELLA Inc, CA) 2 × 10 min (at 40 °C, 150 watts) to aid in antibody penetration. In order to amplify the signal of Iba1 + P2RY12, sections were incubated first in biotinylated donkey anti-rabbit secondary IgG followed by 24 h in streptavidin 635 (1:200, Invitrogen). Autofluorescence was then reduced by incubating brain sections in 10 mM cupric sulfate (50 mM ammonium acetate and 10 mM cupric sulfate in 10 ml deionized water) for 30 min. Before the final mounting and cover-slipping with the Prolong antifade mounting medium, the sections were washed with dH2O and PB to wash off excessive cupric sulfate. Negative control experiments with the primary antibodies omitted were performed on matched sections through the lesion from each case, which yielded no labeling (Additional file 1: Figure S1).

### Intracellular filling during in vitro whole-cell patch-clamp recording and immuno-staining of microglia

To study microglia–spine interactions, we used pyramidal neurons intracellularly filled from whole-cell patch-clamp recordings conducted for our previous study [14]. During Krebs perfusion, a fresh tissue block was harvested from ventral PMC (Fig. 1c) and cut into 300-μm-thick coronal brain slices using a vibratome, which were then placed into room temperature oxygenated (95% O2, 5% CO2) Ringer’s solution. After one hour of equilibration period, individual brain slice was placed in submersion-type recording chambers (Harvard Apparatus), mounted on the stages of Nikon E600 infrared-differential interference contrast microscopes (Micro Video Instruments). Then, in vitro whole-cell patch-clamp experiments and intracellular filling were performed on the layer 3 (L3) pyramidal neurons in the perilesional region at room temperature, as described [14, 34], to obtain electrophysiological data for our previous study [14]. Electrodes were fabricated on a horizontal Flaming and Brown micropipette puller (model P-87, Sutter Instruments) [34]. Potassium methanesulfonate-based solution (concentrations in mM: 122 KCH3SO3, 2 MgCl2, 5 EGTA, 10 Na-HEPES, pH 7.4; Sigma-Aldrich) with 1% biocytin was used as internal solution in electrodes (resistances of 3–6 MΩ) to fill the pyramidal neurons. After recording, slices were fixed in 4% PFA for 2 days. In order to visualize the cells filled with biocytin, slices were incubated in 1% Triton-X in 0.1 M PB for two hours at room temperature, and in streptavidin-Alexa 488 for 2 days (1:500 in 0.1 M PBS, Invitrogen). After recording, slices were fixed in 4% PFA for immunolabeling experiments.

To assess microglia–spine appositions, slices with filled cells were then processed for immunolabeling of microglia. Slices were first incubated in 50 mM glycine for one hour, followed by antigen retrieval with a 20-min incubation in 10 mM citrate buffer (pH = 8.5) at 60–65 °C. To label microglia, a combination of primary antibodies to Iba1 (1:500, Wako, Cat# 019–19741, RRID: AB_839504) and P2RY12 (rabbit, 1:250, Novus Biologicals, NBP2-33,870) was diluted in a carrier solution (0.2% BSA, 1% NDS, 0.1% Triton X-100 in 0.1 M PB). Slices were incubated for 7 days at 4 °C in primary antibodies, with 2 × 10 min low wattage microwave sessions (150 W, 40 °C in Ted Pella biowave) on days 1, 2, 4 and 5, to assist with antibody penetration. Slices were then incubated in anti-rabbit IgG secondary antibody conjugated to Alexa 546 for four days at 4 °C with 2 × 10 min microwave sessions (150 W, 40 °C) performed on days 1 and 3.

### Confocal imaging and quantification of synaptic markers

All 30 or 60-µm sections were imaged by a Leica TCS SPE laser scanning confocal microscope with 3 laser lines: 488 nm, 546 nm, and 647 nm (Leica Microsystems). Sections were imaged with a 40 × 1.3 N.A. oil objective lens at a resolution of 0.134*0.134*0.5 µm. For each section, we imaged four fields, spaced 300-400 µm apart, directly underlying the damaged pial surface (from 200 µm to ~ 1200 µm distal to the damaged pial surface) in M1 gray matter, and two fields in PMC gray matter layers 2/3 with intact pial surface ~ 2 mm distal to the lesion in each brain section (Fig. 1f). Specifically in M1 grey matter we imaged the four fields with the following distances from the lesioned surface: field 1: ~ 200 µm; field 2: ~ 500 µm; field 3: ~ 800 µm; and field 4: ~ 1200 µm. Each confocal image was deconvolved using AutoQuant (Media Cybernetics) and converted to 8-bit images for further analysis.

To quantify the synaptic markers, we analyzed the optical density (percent area labeled) and size of immunolabeled pre- and postsynaptic markers using particle analysis function in FIJI/ImageJ (https://imagej.net/Fiji; 1997–2016; RRID:SCR_002285) [19]. The signal threshold for analysis was set using the Renyi method in FIJI, within field 1 of perilesional M1 and applied to all the other fields of the same section. The physical contacts, or colocalization, between synaptic markers (VGLUT1 & 2, VGAT) and microglial markers (P2RY12 and Iba1) or complement marker C1q were analyzed by using the co-localization plugin of FIJI/ImageJ. The percent area of co-localization was first obtained and then a colocalization coefficient was calculated based on Mander’s method (the percent area colocalized/percent area of marker 1 or marker 2). The average measures of synaptic puncta optical density and microglia–synapse colocalization were calculated for each animal and compared between groups.

### Quantification of microglia–neuron interactions

We assessed the interaction between filled L3 pyramidal neurons and immuno-labeled microglia in the perilesional ventral PMC. Dual channel imaging was conducted using a Leica TCS SPE laser scanning confocal microscope using 488 nm and 546 nm lasers, under 63x/1.4 N.A. oil objective lens, at a resolution of 0.04*0.04*0.3 µm. One apical dendrite and one basal dendrite of each filled cell were followed and scanned from base (point of origin from the soma) to distal tip. Scanned confocal z-stacks  were montaged, and the dendritic segments were traced and reconstructed in Neurolucida 360 (RRID: SCR_016788; MBF Bioscience). The appositions of microglia (P2RY12/Iba1 +) on dendritic shafts and dendritic spines were counted and categorized as  either "contact" or "neighboring" interaction. A "contact" required overlap of saturated signal from the two channels, while "neighboring" interaction was identified when the signal of two channels was adjacent and the distance was ~ 0.3 µm to ~ 1 µm. The traced dendrites with markers of microglial apposition were analyzed and exported using Neuroexplorer (v11.01, Microbrightfield). The density of appositions (# of appositions/total length of dendrite imaged) was calculated for each dendrite.

### Microglia classification and reconstruction

We used the NeuroLucida 360 software (RRID: SCR_016788; MBF Bioscience) to classify and partially reconstruct the somata and proximal processes of microglia. We examined each field through the entire Z-stack and counted the microglia by marking them based on their morphological phenotypes, as described [13, 39]. Based on the continuum of morphologies described in these previous studies, microglia were classified into three categories: 1) ramified microglia were classified based on the appearance of a distinctly round soma, and thin multipolar primary process. 2) Hypertrophic I microglia were characterized by ovoid and slightly enlarged (about 1.5 × larger in diameter than ramified) somata, together with well-ramified but thickened processes (about 2 × thicker than ramified). 3) The amoeboid/hypertrophic II had either large somata (> 2 × larger in diameter than ramified) with very thick and short process, or with highly elongated somata with thick processes almost as thick as the soma diameter. The microglia identified by Iba1 staining and classified by morphology, were then classified based on C1q expression, thus allowing us to quantify six categories of microglial phenotypes as follows: ramified (Rami) C1q-negative cells (R- or Rami-), ramified cells with C1q colocalized in the soma or processes (R+ or Rami+), hypertrophic (Hyper) C1q-negative cells (H- or Hyper-), hypertrophic cells with C1q colocalized in the soma or processes (H+ or Hyper+), amoeboid (Ame) C1q-negative cells (A- or Ame-) and amoeboid cells with C1q colocalized in the soma (A + or Ame+).

Since the established criteria for classification by microglial morphology based on the literature are mainly qualitative [39], the quantitative criteria for defining these distinct microglia subclasses remain unclear. Further, these microglial morphological states represent a continuum and therefore it remains unclear what are the precise multivariate properties that define distinct microglial morphological classes. Thus, we used a bottom-up approach to validate if our top-down user-based classification of microglia via qualitative criteria, corresponded to subclasses with quantitatively distinct morphological features. We  partially 3D reconstructed the soma and primary process of a subset of the classified microglia. Somata were reconstructed in NeuroLucida 360 (Microbrightfield, Inc.), using the 3D environment and soma auto-detection features. The soma detector sensitivity was maintained between 70–90, the interactive search region ranged between 20–30 µm, and the size constraint was between 2–5 µm. For the somata that were not able to be auto-detected , we manually contoured them by using the Cell Body trace feature in the software and focusing through the Z-stack. In addition, all the primary processes of the microglia were manually contoured using the Dendrite feature, since we wanted their thickness to be accurately determined. Once all the microglia were classified and reconstructed, we exported the data for microglia using the NeuroLucida Explorer (v11.01, Microbrightfield).

### RNA isolation and qPCR

Ventral perilesional brain tissue containing the caudal PMC/M1 area of each animal was dissected at euthanasia (14 weeks post-injury), flash-frozen using dry ice, then stored at -80◦C until RNA isolation as described in our previous work [13]. Briefly, tissue samples were thawed on ice and dissected into 100 mg pieces for each animal before mechanical homogenization using an RNAse-free scalpel. Then, tissue samples were chemically triturated using the TRIzol method as follows (ThermoFisher, Waltham, MA): Briefly, tissue was placed in TRIzol and passed through an 18-gauge needle to further homogenize tissue. An organic extraction was then performed using chloroform and ethanol, according to the manufacturer’s protocol (ThermoFisher, Waltham, MA). The extracted RNA was then air-dried and resuspended in 40 µL of PCR-grade water. RNA purity was checked using UV absorbance ratio at A260/280 with a NanoDrop spectrophotometer (ThermoFisher, Waltham, MA).

After extraction, RNA was converted to cDNA using a High-Capacity RNA-to-cDNA kit (ThermoFisher, Waltham, MA), according to the manufacturer’s protocol, then normalized to 2 µg for each sample. Gene expression analysis was performed in triplicate using quantitative polymerase chain reaction (qPCR) with either Taqman Gene Expression Assay or PowerUp Sybr Green Master Mix (ThermoFisher, Waltham, MA). Relative gene expression levels of *GRIA1* (AMPA GLUR1), *GRIA2* (AMPA GLUR2), *GRIN1* (NMDA NR1), *GRIN2B* (NMDA NR2B), *GABRA1* (GABA_a_ α1), *GABRA2* (GABA_a_ α2), *GABRA5* (GABA_a_ α5), *GABRD* (GABA_a_ ∂), *GABBR2* (GABA_b_ R2), *C1qA* (C1q) and C3 (C3) were calculated using the ΔΔCt method with *GAPDH* as reference gene for normalization.

### Statistical analysis

All data were expressed as box-and-whisker plots and vertical scatter plots of individual data points from the three experimental groups: non-lesion control (ctr), lesion with vehicle treatment (veh), and lesion with EV treatment (EV). Statistical analyses were performed in MATLAB (R2020a, MathWorks, Natick, MA) to calculate the average of each field and each animal. For all the box-and-whisker plots, bars represent the interquartile range (lower quartile 25% to upper quartile 75%), with the median indicated by a horizonal line through each bar and the error bars indicating the 95% confidence interval. An outlier analysis, with outliers determined as the value two times the standard deviation from the group mean, was performed for each outcome measure.

For between-group and between-region comparisons, the density of markers in each region was expressed as the average of the four fields sampled in M1 and the two fields sampled in layers 2–3 of dorsal PMC. To compare between-group and between region, a two-way ANOVA with Fisher’s post hoc was used to assess the interactive and independent effects of experimental group and cortical region on all the outcome measures. For pair-wise comparison between groups with lesion, Student’s *t* tests were performed for measures of microglia–spine contacts and qPCR results.

Linear correlations between variables were determined using linear regression analyses in MATLAB. Nonmetric multidimensional scaling (NMDS) was performed to analyze similarities among three groups (ctr, veh, EV) based on 9 gene expression outcome variables from qPCR analyses (*GRIA1*, *GRIA2*, *GRIN1*, *GRIN2B*, *GABRA1*, *GABRA2*, *GABRA5*, *GABRD*, *GABBR2*), and 21 synaptic and microglia outcome measures (%area VGLUT1, VGLUT2, VGAT, GLUR2/3, GABA_a_ α1, GABA_b_ R2; % of VGLUT1, VGLUT2 or VGAT with Iba1; % of Iba1 with VGLUT1, VGLUT2 or VGAT; % area C1q; % of VGLUT2 with C1q; % C1q with VGLUT2; cell densities of ramified, hypertrophic, amoeboid C1q + and C1q- microglia), as described [14]. Z-scores were obtained for each variable, and pair-wise comparisons were used to calculate a distance matrix based on squared Euclidean distances. For qPCR, we clustered the values from individual cases, and for IHC data we clustered the average values from imaging fields in M1 (four fields averaged) and PMC (two fields averaged) separately per case. The multidimensional distance matrix was then reduced to two dimensions via NMDS, and the resulting values of each case were plotted, with the distances between data points representing the relative similarities based on the set of variables. For the results of microglia reconstruction, three-way ANOVA with post hoc Fisher’s LSD was performed in MATLAB for soma volume and aspect ratio, using three factors: group, phenotype/morphology, and C1q ± expression.

## Results

Figure 1a summarizes the experimental workflow as described in more detail in previous work [12–14, 40]. As in our previous studies, the volume of the lesion did not significantly differ between vehicle (mean lesion volume = 33.8 ± 14.9) and EV (lesion volume = 42.4 ± 13.5) treated monkeys (Student’s t = test, t = –0.732, *p = *0.488). However, EV treatment did affect the qualitative appearance of the lesion, specifically with a lighter appearance of lesioned tissue after fixation in EV compared to vehicle monkeys (Fig. 1c). In the current study, we sampled from this cohort adjacent sections through the lesion, matched in rostro-caudal level across the cases, and analyzed 9 markers related to synaptic and microglial function in perilesional M1, the cortex within and around the lesioned area, as well as in the dorsal and ventral PMC, which were more distal to the lesion (Fig. 1c-f).

### Lesion-related reduction of VGLUT2 but not VGLUT1 density in perilesional M1 and PMC

**Fig. 2:**
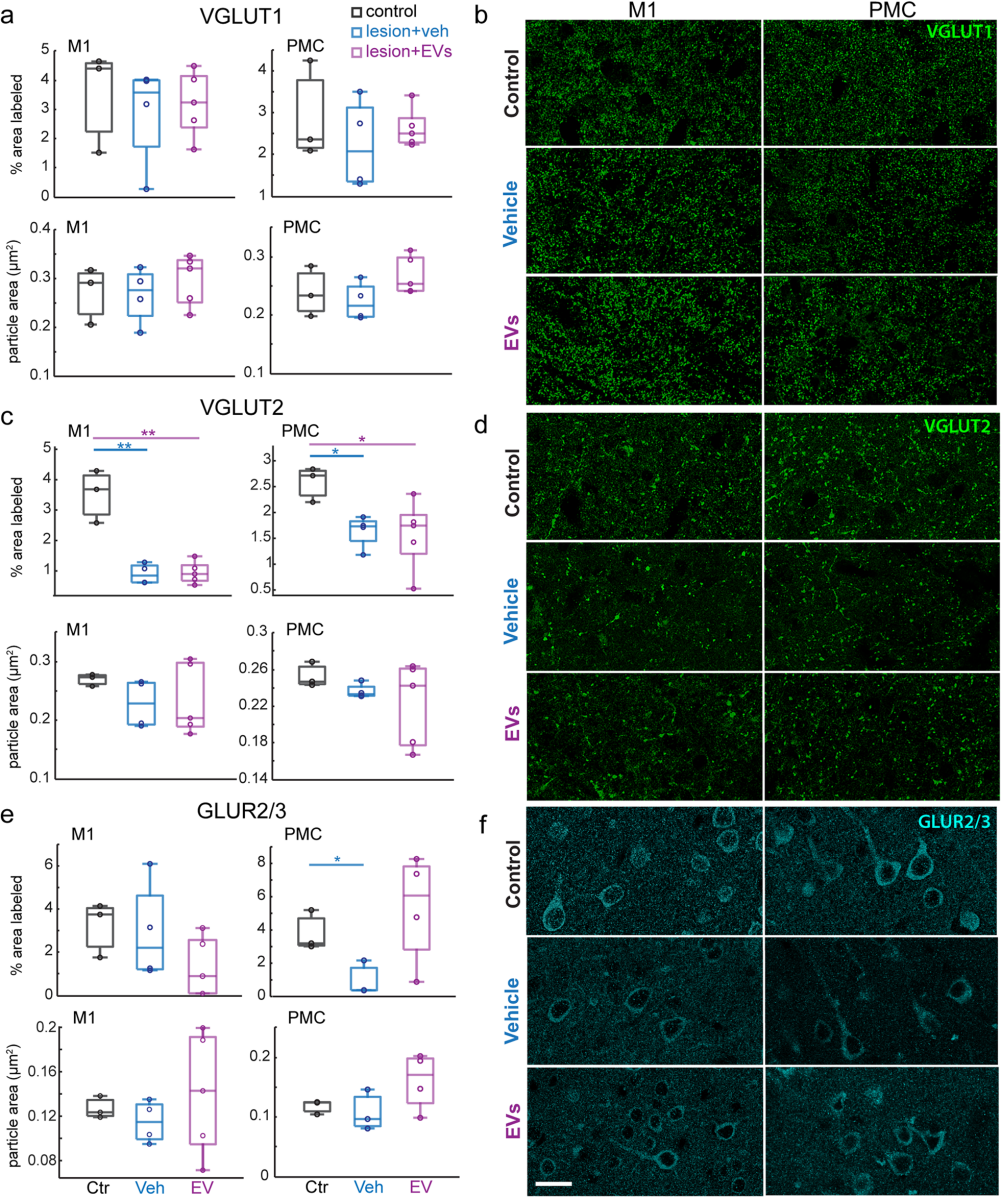
The expression of excitatory synaptic markers in perilesional M1 and PMC. **a** Box-and-whisker plots with vertical scatter plots of individual cases showing the density (% area labeled) and average particle size (area in µm^2^) of VGLUT1 + puncta in perilesional M1 and in PMC of each monkey. **b** Representative confocal images of VGLUT1 immuno-labeling in M1 and PMC. **c** Box-and-whisker plots with vertical scatter plots of individual cases of the density and average size of VGLUT2 + puncta in perilesional M1 and in PMC. The density of VGLUT2 + puncta was significantly lower in groups with lesions compared with Ctr in both perilesional M1 (one-way ANOVA, main effect, *p < *0.001; Fisher’s LSD post hoc, ctr. vs veh: *p = *0.004; ctr vs EV: *p = *0.001) and in PMC (one-way ANOVA, main effect, *p = *0.05; Fisher’s LSD post hoc, ctr. vs veh: *p = *0.04; ctr vs EV: *p = *0.02). **d** Representative maximum-projection confocal images of VGLUT2 immuno-labeling in M1 and PMC. **e** Box-and-whisker plots with vertical scatter plots of individual cases of the density (% area label) and average size (µm^2^) of GluR2/3 + puncta in perilesional M1 and in PMC. The density of GLUR2/3 + puncta in PMC was significantly lower in the vehicle group (one-way ANOVA, main effect, *p = *0.1; Fisher’s LSD post hoc, *p = *0.036) but not in the EV group (Fisher’s LSD post hoc, *p = *0.49) as compared with non-lesion controls. **f** Representative maximum-projection confocal images of GLUR2/3 immuno-labeling in M1 and PMC. For ***a-f*** Ctr: *n = *3. Veh: *n = *4. EV: *n* = 5 monkeys Scale bar: 20 µm. Box-and-whisker plots: bars show interquartile range and median (horizontal line) with error bars = 95% confidence interval; *p < 0.05, **p < 0.01

To assess the extent of synapse loss and remodeling with lesion and EV treatment, we determined the density (% area labeled) and size of immunolabeled presynaptic and postsynaptic structures in perilesional M1 gray matter and perilesional dorsal PMC (Fig. 1d-g). Using one-way ANOVA, we assessed the effect of experimental group (ctr, veh and EV) on the expression of presynaptic axon terminals labeled with VGLUT1 and VGLUT2, which represent two distinct sets of inputs to the cortex. VGLUT1 is known to label mostly terminals from cortico-cortical pathways, while VGLUT2 labels axon terminals from subcortical afferents, mainly cortico-thalamic [41]. We found a significant effect of lesion on the density of VGLUT2 + axon terminals in both perilesional M1 and dorsal PMC (Fig. 2c-d): both EV and veh lesion groups showed significantly lower density (% area labeled) of VGLUT2 + puncta compared to the non-lesioned controls, indicating a lesion-related loss of VGLUT2 in M1 and PMC (Fig. 2c: M1: one-way ANOVA, main effect, *p < *0.001; Fisher’s LSD post hoc, *p < *0.01; PMC: main effect, *p = *0.05; post hoc, *p < *0.05). In contrast to differences in VGLUT2, the VGLUT1 density was not significantly impacted by the lesion (Fig. 2a, b). No significant treatment effect was found for the density of either VGLUT1 or VGLUT2.

### EV treatment mitigated the lesion-related reduction of postsynaptic GLUR2/3 in PMC

In order to assess the effects of lesion and EV treatment on excitatory postsynaptic structures, we analyzed the density of glutamatergic AMPA receptor subunits labeled with GLUR2/3 in perilesional M1 gray matter and PMC. The AMPA receptor is the ionotropic glutamate receptor responsible for fast excitatory synaptic transmission [42]. In PMC, GLUR2/3 subunit density was significantly reduced in the vehicle-treated group compared with non-lesion controls (Fig. 2e: Fisher’s LSD post hoc, *p < *0.05), indicative of a lesion-related decrease in efficacy of AMPA synaptic transmission (Fig. 2e, f). In contrast, the EV treated group did not differ from the non-lesion control group in GLUR2/3 subunit density (Fig. 2e, f). The lesion resulted in a reduction of postsynaptic GLUR2/3 in perilesional cortices, which was ameliorated by EV treatment.

### Lesion-related dysregulation of GABAergic postsynaptic receptor subunit expression in perilesional M1 and PMC

**Fig. 3:**
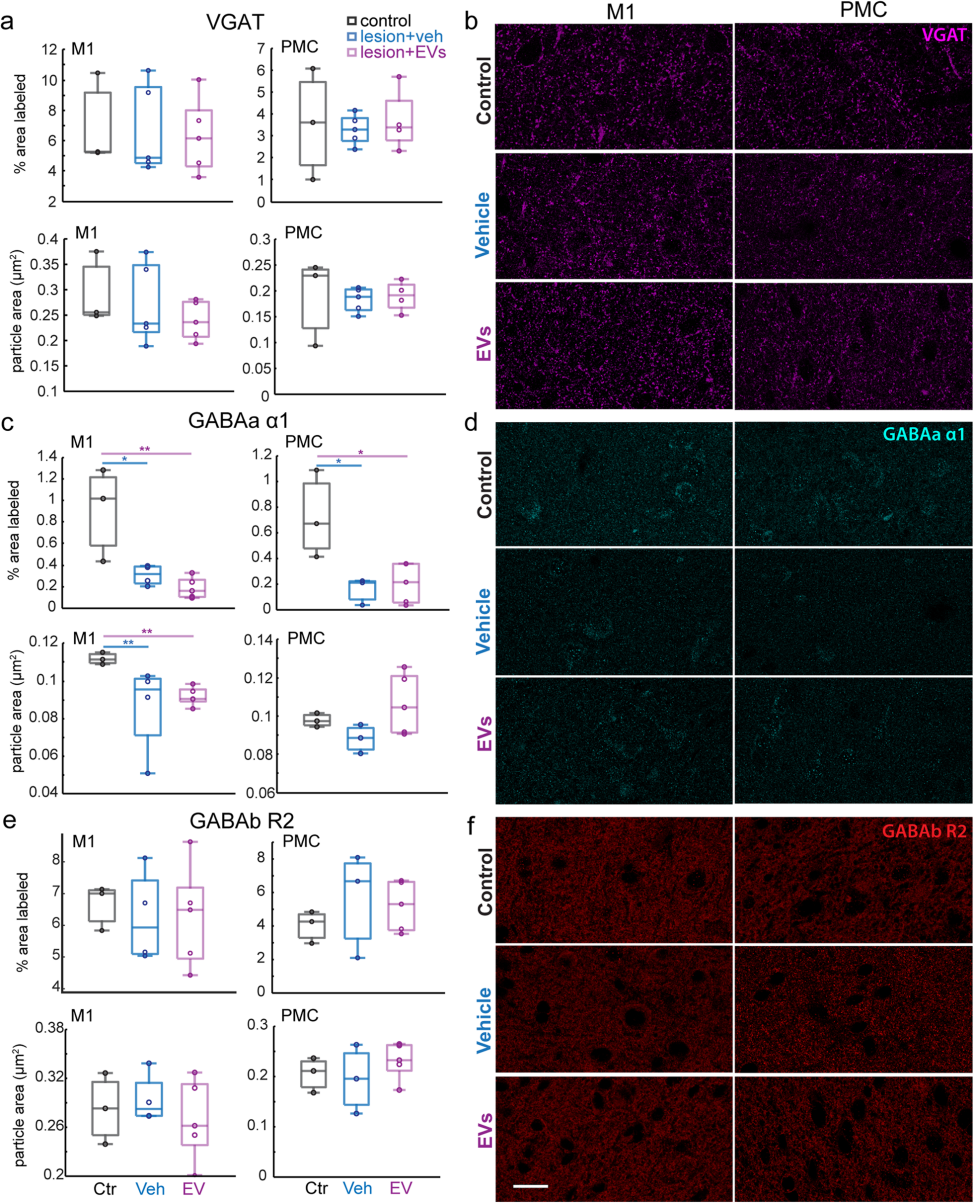
The expression of inhibitory synaptic markers in perilesional M1 and PMC. **a** Box-and-whisker plots with vertical scatter plots of individual cases showing the particle density and average size of VGAT+ puncta in perilesional M1 and in PMC (Ctr: *n* = 3; Veh group: *n* = 5; EV group: *n* = 5 monkeys).** b** Representative maximum-projection confocal images of VGAT immuno-label in M1 and PMC. **c** Box-and-whisker plots with vertical scatter plots of individual cases of the density and average size of GABA_a_ α1+ puncta in perilesional M1 and in PMC. The density of GABA_a_ α1 subunit in perilesional M1 was significantly lower in both veh and EV group as compared with non-lesion controls (one-way ANOVA, main effect, *p = *0.004; Fisher’s LSD post hoc, ctr. vs. Veh, *p = *0.038; ctr. vs. EV, *p = *0.009). The density of GABA_a_ α1 in PMC was lower in veh and EV group (one-way ANOVA, main effect, *p = *0.016; Fisher’s LSD post hoc, ctr. vs. veh, *p = *0.05; ctr. vs. EV, *p = *0.02). The size of GABA_a_ α1 in M1 was significantly smaller in the EV group (*p < *0.001).** d** Representative maximum-projection confocal images of GABA_a_ α1 receptor subunit immuno-label in M1 and PMC. **e** Box-and-whisker plots with vertical scatter plots of individual cases showing the particle density and average size of GABA_b_ R2+ puncta in perilesional M1 and PMC. **f** Representative maximum-projection confocal images of GABA_b_ R2 subunit immuno-label in M1 and PMC. For ***c-f*** Ctr: *n = *3; Veh group: *n = *4; EV group: *n = *5 monkeys. Scale bar: 20 µm. Box-and-whisker plots: bars show interquartile range and median (horizontal line) with error bars = 95% confidence interval; *p < 0.05, **p < 0.01

We assessed the effects of lesion and EV treatment on inhibitory synapses by analyzing the density of immunolabeled presynaptic VGAT axon terminals (Fig. 3a, b) and postsynaptic GABA_a_ α1 (Fig. 3c, d) and GABA_b_ R2 receptor (Fig. 3e, f) subunits. In contrast to the effects of the lesion on excitatory presynaptic terminals, no significant between-group difference was found for presynaptic VGAT in perilesional M1 or PMC gray matter (Fig. 3a, b). However, significant lesion effects were found on the expression of distinct postsynaptic GABA receptor subunits. Post-synaptically located GABA_a_ α1 subunits are associated with ionotropic receptors responsible for fast inhibitory synaptic transmission, whereas GABA_b_ R2 subunits are associated with metabotropic receptors that mediate slow or tonic inhibition [43–45]. There was a significant lesion-related reduction in the density of GABA_a_ α1 + puncta in both perilesional M1 and PMC (Fig. 3c, d: one-way ANOVA, main effect, *p < *0.01; Fisher’s LSD post hoc, M1 and PMC: ctr. vs. veh, *p < *0.05; ctr. vs. EV, *p < *0.05). In perilesional M1, both groups with lesions had smaller average size (area) of GABA_a_ α1 + puncta (Fig. 3c: one-way ANOVA, main effect, *p < *0.001). However, in PMC, the EV-treated group showed a trend for larger size of GABA_a_ α1 puncta than in the vehicle group, which approached significance (Fig. 3c, Fisher’s LSD post hoc, veh vs. EV, *p = *0.1).

### EV treatment normalized gene expression of glutamate and GABA receptor subunits in PMC

**Fig. 4:**
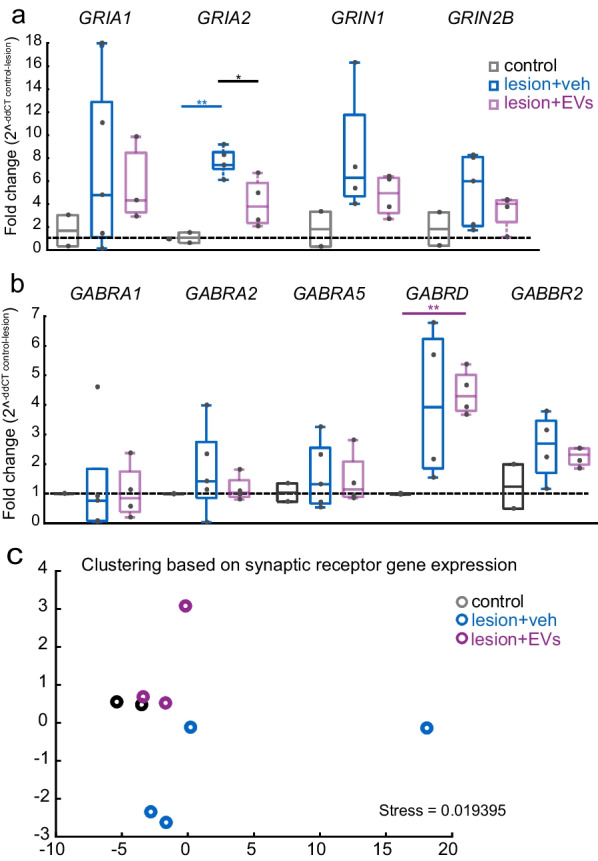
Glutamate and GABA receptors subunit mRNA expression in perilesional cortex. **a** Box-and-whisker plots with vertical scatter plots of individual cases showing relative fold changes in glutamate receptor subunit gene expression. The gene expression of *GRIA2* (GLUR2) was significantly higher in the veh group, as compared with non-lesion controls (*t*-test, *p < *0.001) and the EV group (*p = *0.01). **b** Fold changes of GABA receptor subunit gene expression. The gene expression of *GABRD* (GABA_a_ ∂) was significantly higher in the EV group as compared with non-lesion controls *(t*-test, *p = *0.004). Gene names: *GRIA1* (AMPA GLUR1), *GRIA2* (AMPA GLUR2), *GRIN1* (NMDA NR1), *GRIN2B* (NMDA NR2B), *GABRA1* (GABAa α1), *GABRA2* (GABA_a_ α2), *GABRA5* (GABA_a_ α5), *GABRD* (GABA_a_ ∂), *GABBR2* (GABA_b_ R2). Ctr: *n = *2; Veh: *n = *5; EV: *n = *4 monkeys. **c** MDS plot showing clustering of cases based on mRNA expression profiles of Glu and GABA receptor subunits. The proximity of points indicates the relative similarity-based pair-wise correlation of these multiple mRNA expression variables. Ctr: *n = *2; Veh: *n = *5; EV: *n = *3 monkeys. Box-and-whisker plots: bars show interquartile range and median (horizontal line) with error bars = 95% confidence interval; *p < 0.05, **p < 0.01

We also assessed using qPCR whether the lesion or EV treatment impacted the transcription of glutamate and GABA receptors subunits. Transcript mRNA levels for each gene were expressed as fold-change relative to average of non-lesion controls, and the two lesion groups were compared using pair-wise *t*-test. Outlier analyses were performed and some data points were excluded based on atypically high values of delta-CT. Our results showed a lesion-related increase in *GRIA2* (AMPA GLUR2) mRNA expression, which was reduced by EV treatment (Fig. 4a, Student’s *t*-test; ctr vs. veh: *p < *0.01; veh vs. EV: *p < *0.05). A similar pattern of expression was found for *GRIN1* (NMDA NR1) which approached significance between control and vehicle group (Fig. 4a, ctr vs. veh: *p = *0.06, con vs. EV: *p = *0.14).

Interestingly, our results showed a lesion-related increase in gene expression of *GABBR2* (GABA_b_ R2) and *GABRD* (GABA_a_ ∂), which transcribe GABA receptor subunits that mediate tonic inhibitory currents (Fig. 4b, p < 0.01). To assess (dis)similarities across cases based on these multivariate mRNA expression profiles of synaptic markers, we performed dimensional reduction and clustering using NMDS (Fig. 4c), as described [14]. Each outcome measure was normalized as z-scores, and pair-wise comparisons across all subjects were employed to create a distance matrix based on between-subject squared Euclidean distances. For qPCR, we clustered the individual cases based on this distance matrix from comparisons of the mRNA levels of 9 genes of the 9 subjects with complete sets of outcome measures (the four samples where data points were missing were excluded). The multidimensional distance matrix was used to plot NMDS and show clustering of individual cases. The NMDS plot showed that veh and EV-treated monkeys formed distinct clusters (Fig. 4c). Further the EV-treated cluster overlapped with non-lesion control, indicating that EV treatment shifted gene expression of GLURs and GABARs towards the non-lesion control expression pattern. In contrast, vehicle-treated lesion monkeys were more dissimilar (clustered farther in the NMDS plot) to non-lesion controls based on GLURs and GABARs gene expression. These data suggest that EV treatment results in a “normalization” of lesion-related changes in gene expression of GLURs and GABARs.

### Lesion-related increase in microglia interaction with synaptic elements

**Fig. 5:**
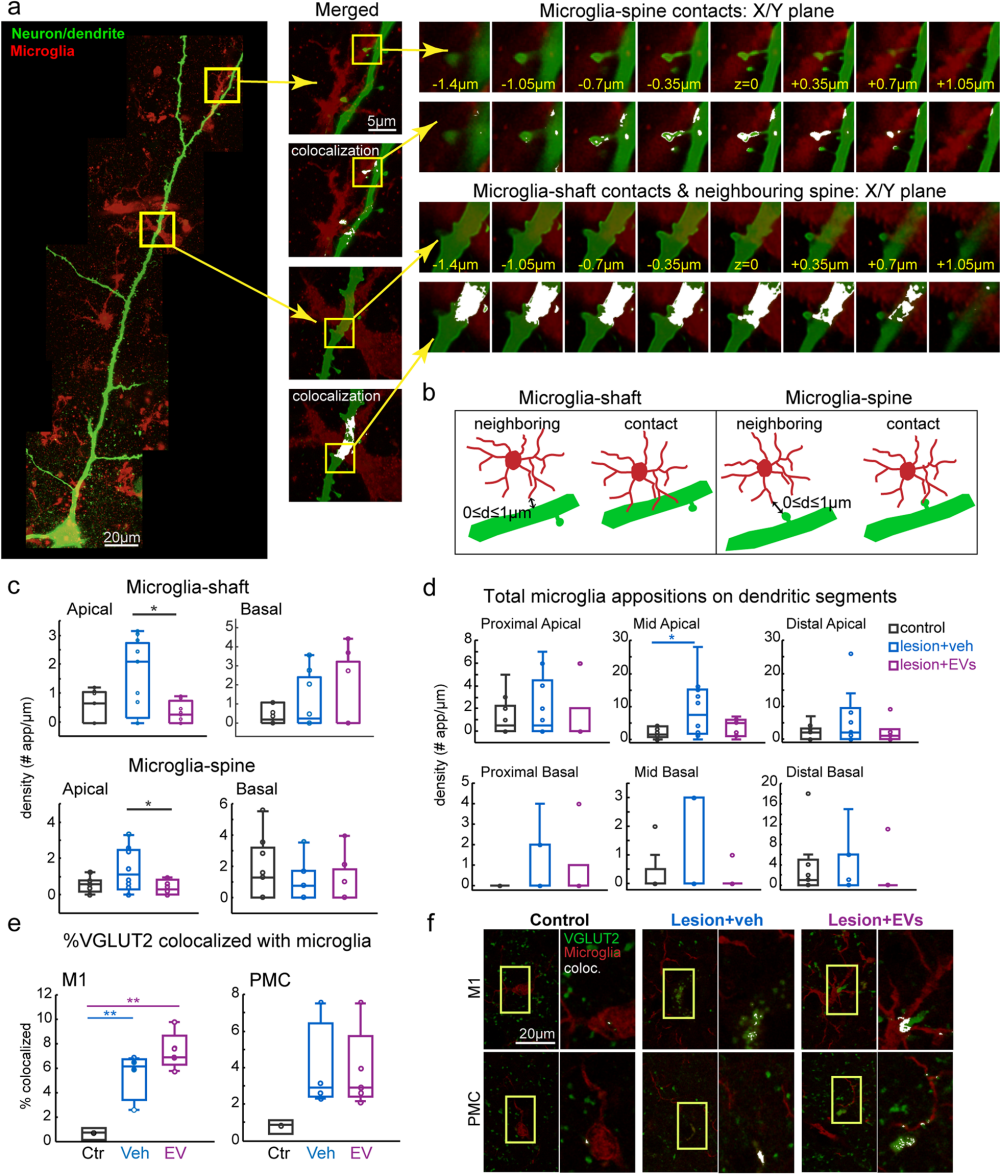
Microglia apposition on synaptic structures. **a** Representative images of microglia interactions with dendritic spines/shaft at different z-levels of stack. Neuronal dendrites and spines were filled with biocytin and stained with streptavidin-Alexa 488 (green) and microglia were visualized with Iba1 and P2RY12 + (red) immuno-labeling. White pixels indicate an overlap between two channels. Scale bars: 20 µm and 5 µm. **b** Schematic diagram of criteria for determining microglia appositions on dendritic shafts or spines, classified as either contact (touching) or neighboring (within 1 microns).** c** Box-and-whisker plots with vertical scatter plots of individual neurons showing the density of microglia appositions (contacting and neighboring) on dendritic shaft and spine. The vehicle group had higher densities of microglial–apical shaft (one-way ANOVA, main effect, *p = *0.07; Fisher’s LSD post hoc, veh vs. EV, *p = *0.009) and microglial–apical spine (*p = *0.038) appositions compared to EV group, and a trend for greater total appositions in the vehicle than in the control group was found (ctr. vs. veh., *p = *0.058). **d** Total microglial appositions (contacts and neighboring on spines and shafts) on different segments of apical/basal dendrites in ventral PMC. Mid-apical dendrites had higher densities of microglial contacts only in veh compared to control (ctr. vs veh, *p = *0.03; ctr. vs EV, *p = *0.84). For ***c-d*** Ctr: *n = *10 cells from 3 monkeys; Veh group: *n = *10 cells from 3 monkeys; EV group: *n = *7 cells from 2 monkeys.** e** Box-and-whisker plots with vertical scatter plots of individual cases showing the fraction of VGLUT2 colocalized with microglia, which was higher in both groups with lesions in M1 compared to control group (ctr. vs. veh, *p = *0.009; ctr. vs. EV, *p < *0.001). Ctr: *n = *3; Veh group: *n = *4; EV group: *n = *5 monkeys.** f** Representative images of dual channel labeling of microglia (red) and VGLUT2 (green), with right panel showing the inset (yellow box) at higher resolution, with colocalized VGLUT2–microglia label masked in white. Box-and-whisker plots: bars show interquartile range and median (horizontal line) with error bars = 95% confidence interval; *p < 0.05, **p < 0.01

Based on our finding of lesion-related decreases in excitatory VGLUT2 + presynaptic axon terminals (Fig. 2c, d) and postsynaptic GLUR2/3 expression (Fig. 2e, f), we then investigated the role of microglia in the processes of synaptic remodeling with lesion and EV treatment. Complete overlap of microglia and synaptic elements can be indicative of actual phagocytosis. However, microglia closely apposed or proximal to synaptic elements can also indicate microglia–synapse signaling to initiate phagocytosis, release of neurotrophic factors or enhancement of plasticity [4, 27]. First, we assessed microglia interactions on the post-synaptic structures (dendritic shaft and spines), by quantifying close appositions (including "contacts" and "neighboring" interactions) between Iba1/P2RY12 + microglial processes and dendrites of intracellularly filled L3 pyramidal neurons in ventral PMC (Fig. 1c, e, f; Fig. 5a). A microglia–dendrite apposition was defined as a “contact” when a microglial process directly overlaps with the dendritic spine or shaft, or as a close “proximity/neighboring” interaction when a microglial process was within one micron from the labeled neuronal dendritic spine or shaft (Fig. 5a, b). For the total microglia–dendrite appositions, which includes both direct contacts and neighboring interactions between microglia and dendrites (shafts and spines), the proportion of microglial appositions with spines versus shafts were about equal (microglia–spine: ~ 40–60%). However, both microglia–spine and microglia–shaft interactions exhibited significant between-group differences, specifically on apical but not basal dendrites (Fig. 5c). Apical dendrites from neurons in vehicle group exhibited significantly higher density [#of appositions/100 µm dendrite length] of microglia–shaft (one-way ANOVA, main effect, *p = *0.07; Fisher’s LSD post hoc, *p < *0.01) and microglia–spine (*p < *0.05) interactions than those from neurons in EV group (Fig. 5c). Further, when looking at specific compartments, we found that compared with non-lesion control group, the mid-apical dendritic segments had significantly higher density of total microglia appositions for pyramidal neurons in vehicle (ctr. vs veh, *p < *0.05) but not in EV group (ctr. vs EV, *p = *0.84; Fig. 5d).

We then assessed the overlap of markers of microglia, Iba1 and presynaptic VGLUT2 + axon terminals. The fraction of VGLUT2 colocalized with Iba1 was greater in both groups with lesion in perilesional M1 (Fig. 5e, f, Fisher’s LSD post hoc*,* *p < *0.01) compared to non-lesion controls. These results suggest that the lesion was associated with increased microglial appositions on presynaptic VGLUT2 + terminals and postsynaptic structures (dendrites and spines) in perilesional motor cortices, and EV treatment reduced the lesion-related increase in microglial contacts on apical dendrites.

### Lesion-related increase in C1q complement receptor expression on VGLUT2+ axon terminals and microglia

**Fig. 6:**
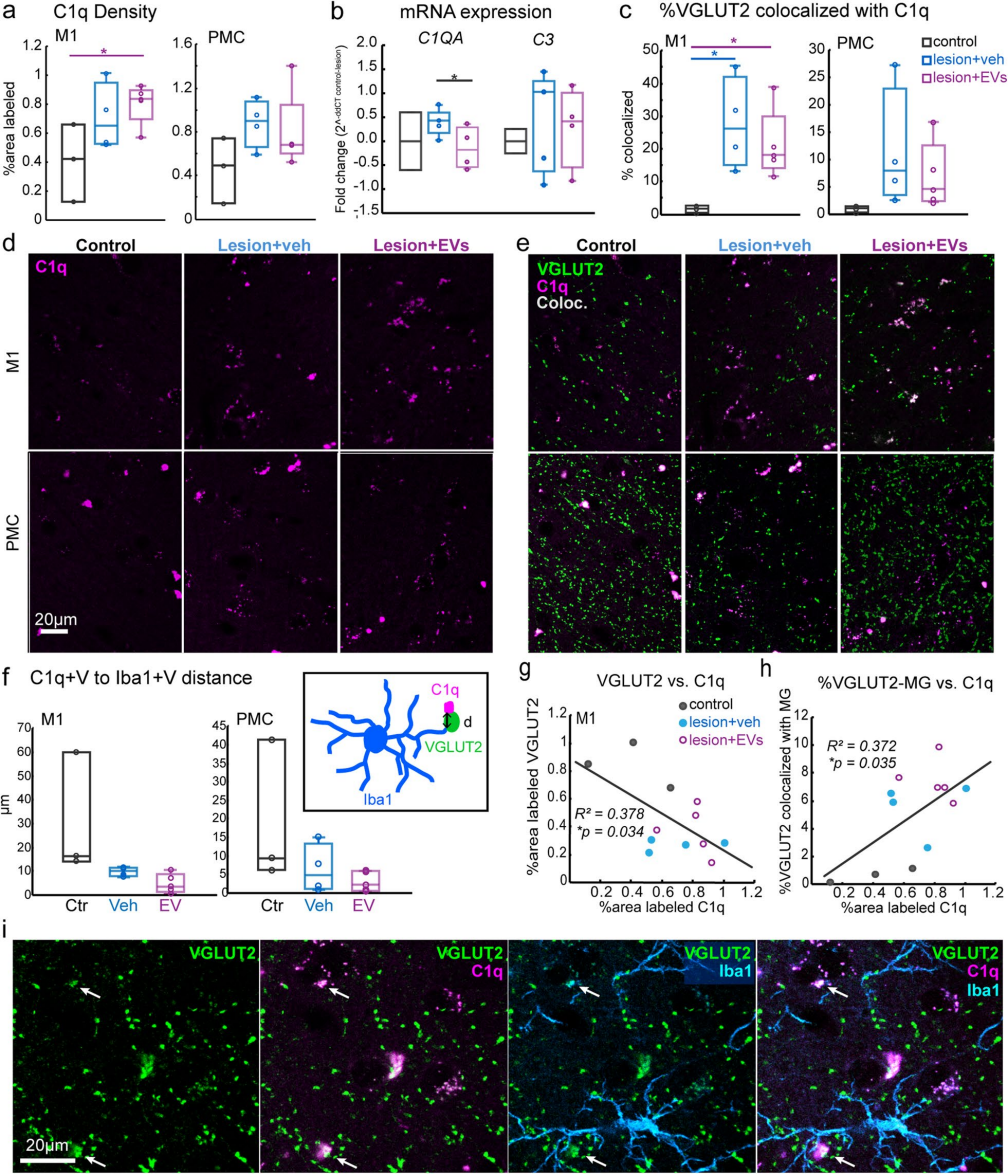
C1q co-expression on VGLUT2 + axon terminals and Iba1 microglia. Box-and-whisker plots with vertical scatter plots of individual cases showing: **a** The density (% area label) of C1q + puncta in perilesional M1 and PMC (one-way ANOVA, main effect, *p = *0.07; Fisher’s LSD post hoc, M1: ctr. vs EV *p = *0.028). Ctr: *n = *3. Veh: *n = *4. EV: *n = *5 monkeys. **b** Fold changes of *C1QA* and *C3* gene expression in perilesional M1 (*t*-test, veh. vs. EV, *p = *0.027; Ctr: *n = *3. Veh: *n = *4. EV: *n = *4). Ctr: *n = *3; Veh: *n = *4; EV: *n = *4 monkeys; and **c** the density (% area label) of VGLUT2 + puncta colocalized with C1q + puncta (one-way ANOVA, main effect, *p = *0.03; Fisher’s LSD post hoc, ctr. vs. veh, *p = *0.02). Ctr: *n = *3; Veh: *n = *4; EV: *n = *5 monkeys. **d** Representative maximum-projection confocal images of C1q + puncta immuno-labeling in M1 and PMC. Scale bar: 20 µm. **e** Representative maximum-projection confocal images showing dual channel labeling of C1q (magenta) and VGLUT2 (green), with colocalized VGLUT2-C1q points masked in white. **f** Box-and-whisker plots with vertical scatter plots of individual cases showing the distance between the C1q-VGLUT2 colocalized points and microglia (Iba1)-VGLUT2 colocalized points. Inset shows schematic diagram of how C1q-VGLUT2-Iba1 distance was determined. Ctr: *n = *3; Veh: *n = *4; EV: *n = *5 monkeys.** g** Linear regression showing increasing C1q expression correlated with decreasing expression of VGLUT2 + (*R*^*2*^ = 0.378, *p = *0.034) in M1. **h** Linear regression showing greater C1q expression correlated with greater VGLUT2–microglia colocalization (*R*.^*2*^ = 0.372, *p = *0.035).** i** Representative maximum-projection confocal images of 9 optical slices showing single/dual/triple channel labeling (left to right) of colocalized VGLUT2 (green), Iba1 (cyan) and C1q (magenta). Box-and-whisker plots: bars show interquartile range and median (horizontal line) with error bars = 95% confidence interval; *p < 0.05

The finding of lesion-related reduction of pre- (VGLUT2) and postsynaptic (GLUR2/3) markers coupled with a lesion-related increase in microglia appositions with these synaptic elements suggested a role of microglia in synapse phagocytosis and/or pruning. C1q, as a complement protein, contributes to synapse elimination by initiating the classical complement cascade [46]. In particular, C1q tags apoptotic cells or cellular debris, including damaged synapses, and triggers downstream signaling for phagocytic elimination by macrophages or microglia [28, 46]. We immuno-labeled C1q to further assess whether the lesion-related increase in these presumed “microglia–synapse interaction” is associated with synapse phagocytosis. In perilesional M1, the density of C1q + puncta was significantly higher in the EV group than in the non-lesion control group (Fig. 6a, d, M1; one-way ANOVA, main effect, *p = *0.07; Fisher’s LSD post hoc, ctr. vs. EV: *p < *0.05) while no significant between-group difference was found in PMC. Similarly, *C1QA* transcript levels in the ventral perilesional M1 was significantly greater in EV compared to vehicle group (Fig. 6b, qPCR fold change, *t*-test, veh. vs. EV, *p < *0.05). Interestingly, no significant between-group differences were found with regard to mRNA transcript levels of *C3*, a downstream target of C1q. Overall, these data suggest an upregulation of C1q activity near the lesion, which was enhanced by EV treatment. This C1q upregulation was not associated with a downstream upregulation of C3 receptor pathway, suggesting involvement of a different complement pathway cascade. Further, it is unclear if this EV-mediated C1q upregulation reflects increased C1q tagging of damaged synapses or increased C1q expression within microglia.

To estimate the synapses tagged with C1q, we assessed the colocalization between VGLUT2 + and C1q + puncta (Fig. 6c, e). We found a significant lesion-related increase in the fraction of VGLUT2 + puncta colocalized with C1q + in M1 in both treatment groups (Fig. 6c, e, one-way ANOVA, main effect, *p < *0.05; Fisher’s LSD post hoc, ctr. vs. veh and ctr. vs. EV, *p < *0.05). In M1, about 20–40% of all VGLUT2 + puncta were expressing C1q in the lesion groups, compared to the non-lesion group where only virtually no VGLUT2 + puncta expressed C1q. This lesion-related increase in the fraction of VGLUT2 + tagged with C1q was not significant in PMC (Fig. 6c, e; ctr. vs. veh, *p = *0.17; con. vs. EV, *p = *0.15), suggesting that C1q tagging of presynaptic VGLUT2 + terminals was prevalent within the M1 area most proximal to the lesion, and was diminished in areas more distal to the lesion. We then assessed whether these C1q + VGLUT2 + puncta were associated with and were within the vicinity of microglia-VGLUT2 contacts (Fig. 6f, i). Thus, we estimated the distance between C1q + VGLUT2 + puncta and microglia Iba1 + VGLUT2 (C1q + V-Iba1 + V distance) contacts (Fig. 6f). We found in M1 but not PMC, a trend of shorter C1q + V-Iba1 + V distance in EV group as compared to the control and vehicle groups (Fig. 6f, M1: ctr. vs. EV and veh. vs. EV, *p = *0.06). These results suggested that within perilesional M1, microglial contacts on VGLUT2 + synapses were associated with the C1q tagging on these terminals, which implied an EV-related upregulation of microglial phagocytosis of VGLUT2 + synapses within the area nearest to the lesion.

### Relationship of lesion-related VGLUT2 loss to microglia C1q expression

Given that we found decreased synaptic marker expression and corresponding increased VGLUT2-C1q colocalization and microglia interaction associated with lesion, we therefore used linear regression to assess relationships between C1q and VGLUT2 densities, and VGLUT2, C1q and microglia (MG) co-localization (Fig. 6g–i). In M1, the decreased density of VGLUT2 + puncta was associated with increased density of C1q + puncta (Fig. 6g, R^*2*^ = 0.378, *p < *0.05), and increased fraction of VGLUT2 tagged by C1q (not shown,* R*^*2*^ = 0.536, *p < *0.01). Further, increased microglia-VGLUT2 co-localization in M1 was associated with increasing density of C1q + puncta (Fig. 6h, R^*2*^ = 0.372, *p < *0.05), shorter distance between C1q tag and microglia-VGLUT2 contacts (not shown,* R*^*2*^ = 0.474, *p < *0.05), and more C1q-Iba1 colocalization (not shown,* R*^*2*^ = 0.725, *p < *0.001). These data together indicated that the higher expression of C1q and increased C1q tagging of VGLUT2 + in perilesional M1 was associated with loss of VGLUT2 + and increased VGLUT2-C1q-microglia interaction after lesion, which implicated an increase in microglial synaptic phagocytosis.

### EV treatment increased C1q expression in hypertrophic microglia

**Fig. 7:**
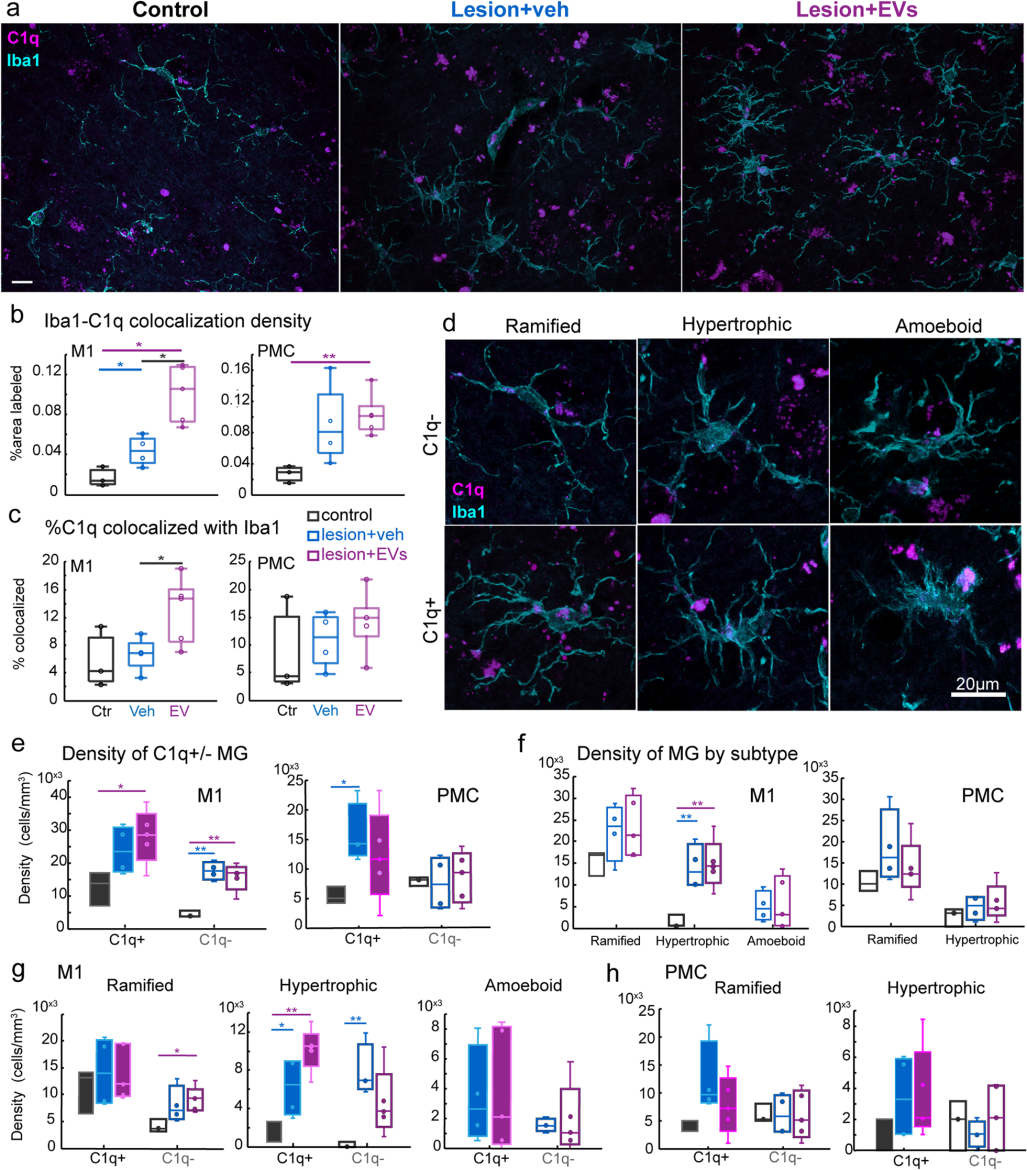
C1q expression in different microglia phenotypes. **a** Representative images of dual labeling of microglia (Iba1, cyan) and C1q (magenta) in all three groups. **b-c** Box-and-whisker plots with vertical scatter plots of individual cases showing: **b** the density (% area label) of Iba1 + colocalized with C1q + puncta. (M1: one-way ANOVA, main effect, *p = *0.001; Fisher’s LSD post hoc, ctr. vs. veh, *p = *0.046; ctr. vs. EV, *p = *0.003; veh vs. EV, *p = *0.009; PMC: main effect, *p = *0.04; post hoc, ctr. vs. EV *p = *0.004); **c** the fraction of C1q + puncta colocalized with microglia (Iba1). Higher fraction of C1q + puncta colocalized with microglia (Iba1) in EV than in veh (main effect, *p = *0.06; post hoc, *p = *0.05). For ***b-c,*** Ctr: *n = *3. Veh: *n = *4. EV: *n = *5 monkeys. **d** Representative images of dual labeling of microglia (Iba1, cyan) and C1q (magenta), with examples of different microglia morphologies with or without C1q. **e–h** Box-and-whisker plots with vertical scatter plots of individual cases showing the cell densities of: **e** microglia by C1q expression. In M1, EV but not veh had greater C1q + microglia density than control (two-way ANOVA group*area, main effect, ‘group’: *p = *0.009; post hoc*:* ctr. vs. veh: *p = *0.07; ctr. vs. EV: *p = *0.03); and C1q- microglia had greater density in lesion than in controls (main effect, ‘group’: *p = *0.006; post hoc*:* con. vs. veh: *p < *0.001; ctr. vs. EV: *p = *0.004). In PMC, greater density of C1q + microglia in veh than control group (*p = *0.02). **f** Microglia by morphology (Rami, Hyper and Ame; two-way ANOVA group*area). In M1, hypertrophic microglia had greater density in lesion compared to control group (main effect, ‘group’: *p = *0.003; post hoc*:* con. vs. veh: *p = *0.01; con. vs. EV: *p = *0.008). **g** Microglia by both C1q expression and morphology in M1. In M1, the EV group showed higher density of C1q- ramified microglia than control group (*p = *0.01). Both groups with lesion had higher density of hypertrophic C1q + microglia than controls (main effect, ‘group’: *p < *0.001; post hoc*:* ctr. vs. veh: *p = *0.047; ctr. vs. EV: *p < *0.001). However, only veh group had a higher density of hypertrophic C1q- microglia than controls (main effect, ‘group’: *p = *0.036; post hoc*:* *p = *0.005). **h** Microglia by both C1q expression and morphology in PMC revealed no between-group differences. For ***e–h*** Ctr: *n = *3; Veh: *n = *4; EV: *n = *5 monkeys. Box-and-whisker plots: bars show interquartile range and median (horizontal line) with error bars = 95% confidence interval; *p < 0.05, **p < 0.01

While we found a lesion effect on C1q tagging of VGLUT2 + excitatory boutons, we did not find EV treatment effects on C1q expression and VGLUT2 tagging. Since microglia produce and store C1q [26], as well as phagocytose C1q tagged debris, we therefore assessed the Iba1-C1q colocalization. We found a lesion-related increase in the total density (percent area) of Iba1-C1q colocalization in M1 in both treatment groups (Fig. 7b, one-way ANOVA, main effect, *p < *0.01; Fisher’s LSD post hoc, ctr. vs. veh, and ctr. vs. EV, *p < *0.05). In PMC, only the EV group exhibited a significant increase in Iba1-C1q colocalization density compared to non-lesion control (Fig. 7b, p < 0.01). Further a treatment effect was found in M1, with EV treated monkeys having significantly greater fraction of C1q puncta colocalized with Iba1 compared to vehicle group (Fig. 7c, p < 0.05).

Given the findings from our previous study that EV treatment promoted a morphological shift from inflammatory to homeostatic ramified microglia [13], we therefore assessed whether this increased C1q + expression in microglia was associated with specific microglial morphologies that are thought to reflect distinct immune activation states [39]. Ramified microglia are characterized by small round somata, thin and highly branched processes, thought to be in a surveilling homeostatic state. Upon immune activation, microglia transition toward an amoeboid state, with an enlarged somata and a few short and thick processes thought to reflect a phagocytic state. A transitional state between ramified and amoeboid states are hypertrophic microglia that have intermediate thick process, more polarized or ovoid intermediate sized somata, and branching can be extensive or not depending on state of transition. Molecular identification of microglia specifically in the ‘polarized’ hypertrophic states suggest distinct sub-populations that have either downstream anti-inflammatory or pro-inflammatory effects [36].

We classified Iba1 + microglia based on their C1q expression (C1q + vs C1q-) and their morphologies—ramified (Rami), hypertrophic (Hyper), or amoeboid—and quantified the densities of these microglia subtypes in perilesional M1 and PMC (Figs. 7d). In both veh and EV lesion groups, there was a greater density of C1q- microglia as compared to the non-lesion control group in M1 (Fig. 7e, two-way ANOVA group*area, main effect, ‘group’, *p < *0.01; Fisher’s LSD post hoc: ctr. vs. veh and ctr. vs. EV, *p < *0.01). However, we found regional and treatment-related differences specifically in the density of C1q + microglia. Specifically, consistent with particle analyses data, the EV but not veh group had significantly greater density of C1q + microglia in M1 as compared to the non-lesion controls (Fig. 7e, post hoc: ctr. vs. EV, *p < *0.05). Conversely, in PMC, the veh but not EV group had a higher density in C1q + microglia compared to the controls (Fig. 7e, ctr. vs. veh, *p < *0.05).

Assessments of total Iba1 + microglia by morphological subtype revealed a lesion-related increase in the density of hypertrophic microglia of both veh and EV groups only in M1 (Fig. 7f, ctr. vs. veh and ctr. Vs. EV, *p < *0.01). This lesion-related increase in hypertrophic microglia in M1 is due both C1q + and C1q- subpopulations in the veh monkeys (ctr. vs. veh, C1q + *p < *0.05, C1q- *p < *0.01), but only due to the C1q + subpopulation in the EV monkeys (ctr. vs. EV, *p < *0.01; Fig. 7g). Further in M1, the EV group but not veh group had a higher density of C1q- ramified microglia than control group (Fig. 7g, p < 0.05). No significant between-group differences in the densities of microglia subtypes were found in PMC (Fig. 7h).

**Fig. 8:**
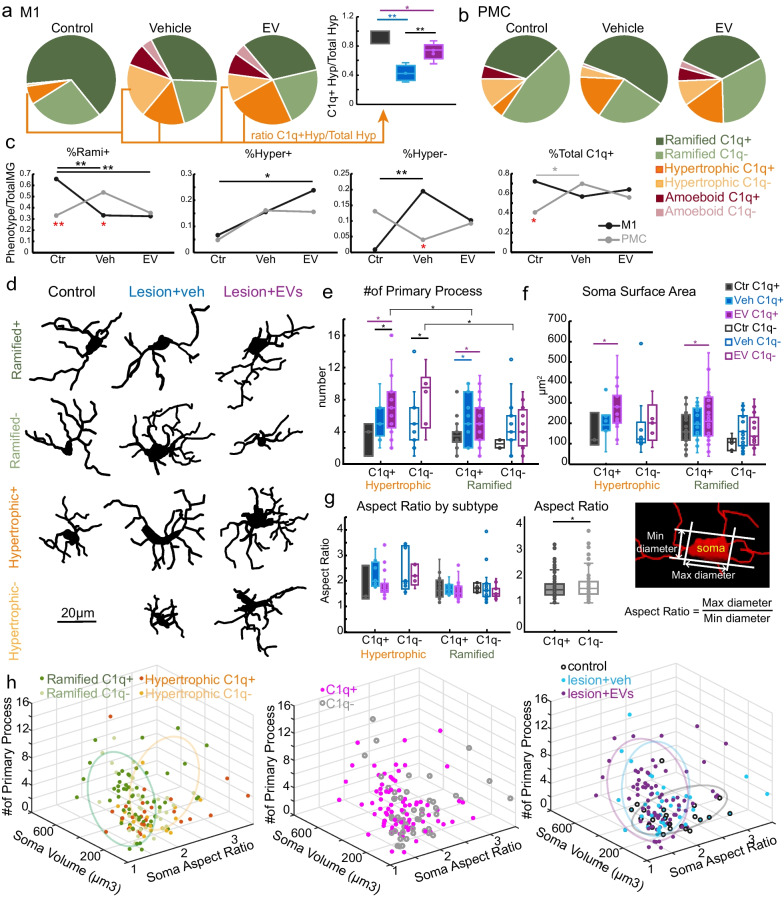
The expression of different microglia phenotypes in M1 and PMC. **a** Pie charts of %microglia by morphology and C1q expression in M1 and **b** PMC. Right inset in ***a*** shows a Box-and-whisker plot of the ratio of Hyper + to the total Hyper microglia (two-way ANOVA group*area: interaction, *p < *0.01; Fisher’s LSD post hoc, *p < *0.05, see Table S3). **c** Line plots showing the mean relative proportion of Rami + , Hyper + , Hyper-, and Total C1q + as a percentage of total microglia in M1 and PMC (two-way ANOVA: main effect ‘group’ %Hyper + ; ‘group*area’ interactions %Rami + , %Hyper-, and %Total C1q + , *p < *0.05). *Between-area* comparisons: in control, %Rami + and %C1q is greater in M1 than PMC; and in vehicle, %Rami + is greater in PMC than in M1, but %Hyper- is greater in M1 than in PMC (Fisher’s LSD, *p < *0.05; Ctr: *n = *3; Veh: *n = *4; EV; *n = *5 monkeys). **d** Representative reconstructions of microglia morphological subtypes. **e–g** Morphological parameters measured from partial 3D reconstructions (Ctr: *n = *36 cells; Veh: *n = *58 cells; EV: *n = *71 cells each from 1 monkey):** e** number of primary process (three-way ANOVA group*morphology*C1q ± , main effect ‘morphology’: *p = *0.02; ‘group’: *p = *0.01; Table S3). *Between-group* comparisons: Hyper ± microglia in EV had more primary processes than in veh/ctr. *Between-morphology* comparisons: In EV, Hyper ± microglia had more primary processes than Rami ± microglia (Fisher’s LSD, *p < *0.05). **f** Microglia soma surface area (three-way ANOVA, main effect ‘group’: *p = *0.006; ‘C1q ± ’: *p = *0.004). *Between-group* comparisons: Hyper + and Rami + microglia in EV had greater soma surface area than in ctr (*p < *0.05). **g** Soma aspect ratio was greater in C1q- microglia than in C1q + (main effect ‘C1q ± ’: *p = *0.03). Left panel shows plots by microglia morphology and C1q subtype. Middle panel shows plot by total C1q+ vs C1q- microglia. Right panel shows example image with aspect ratio measurement. **h** 3D scatter plot of morphological parameters. Annotations based on morphology, C1q expression, and experimental group. Box-and-whisker plots: bars show interquartile range and median (horizontal line) with error bars = 95% confidence interval; *p < 0.05, **p < 0.01. Table S3 for exact p-values

We then assessed whether the density and relative proportion of each microglia phenotype based on morphology and C1q expression were altered by lesion and treatment. Importantly, two-way ANOVA of experimental group*area showed that lesion-related shifts in the distribution of microglia subtypes by C1q expression and morphology were region-dependent (density of C1q- microglia, ‘group*area’ interaction, *p < *0.01). Between-group comparisons showed that in M1 but not PMC there was a lesion-related increase in density of total and C1q + (Hyper +) hypertrophic microglia (Fig. 7e, f; Fisher’s LSD post hoc*,* *p < *0.05 for all comparisons). This group*area interaction effect was also found in the proportions of microglia subtypes, indicating that lesion differentially shifted the relative proportions of microglia subtypes in a region-dependent matter (Fig. 8a–c, two-way ANOVA, ‘area*group’ interaction, %Rami + : *p < *0.01; %Hyper−: *p < *0.05; %C1q + : *p < *0.05). Specifically, M1 but not PMC exhibited lesion-related reduction of % C1q + ramified (Rami +) microglia (Fig. 8c, ctr. vs. veh. and ctr. vs. EV: *p < *0.01), and lesion-related increase in % C1q- hypertrophic (Hyper-) microglia (Fig. 8c, ctr. vs. veh.: *p < *0.01). Interestingly, there was an EV treatment effect on the proportion of C1q + vs C1q- hypertrophic microglia, which also varied based on region. Specifically, two-way ANOVA results demonstrated that the EV treated group but not the veh group had a significantly higher proportion of hypertrophic C1q + microglia in M1 as compared to the controls (Fig. 8c, ctr. vs. EV: *p < *0.05). In contrast, there was a greater proportion of hypertrophic C1q- microglia in the M1 of the veh group compared to non-lesion controls (Fig. 8c, ctr. vs. veh, *p < *0.05).

Given that we found between-group differences in hypertrophic microglia, we assessed the ratio of C1q + hypertrophic microglia to total hypertrophic microglia in M1 for all three groups (Fig. 8a, box-and-whisker plots). Our results showed that in M1 of non-lesion control monkeys, almost all (94%) hypertrophic microglia were C1q + , and this proportion was greater compared to the groups with lesion (Fig. 8a, Hyper+/Total Hyper post hoc: ctr. vs. veh, *p < *0.01; ctr vs. EV, *p < *0.05). EV treatment attenuated this lesion-related proportional decrease in C1q + hypertrophic microglia. In the vehicle group, about 40% of hypertrophic microglia were C1q + ; in the EV group, this proportion was significantly greater, with about 70% of hypertrophic microglia expressing C1q + (Fig. 8a, Hyper + /Total Hyper post hoc: veh vs. EV, *p < *0.01).

### Lesion and EV treatment affects region-specific expression of microglia phenotypes

The data above indicated that EV treatment affected specifically hypertrophic C1q + expression in M1. However, EV treatment seemed to also affect the lesion-related shifts in between-area differences in the distribution of microglial subtypes in M1 versus PMC. Notably, in non-lesion control, a between-area difference was found in the density and proportion of C1q + ramified and hypertrophic microglia. A two-way ANOVA revealed a main effect of cortical 'area' on the density of different microglia subtypes (Fig. 7e–h, two-way ANOVA, main effect, ‘area’, *p < *0.05 for all subpopulations), indicating that microglia phenotypes were expressed differently in M1 and PMC. In M1 of the non-lesion control, majority of both ramified and hypertrophic microglia were C1q + . In PMC of the non-lesion control, majority of hypertrophic microglia were C1q- and a more equal distribution of C1q + and C1q- ramified microglia was found (Fig. 8a, b). In non-lesion control, M1 had significantly greater density of Rami + , Hyper + and Hyper- microglia than PMC (Fig. 7g, h, p < 0.05 for all comparisons). Further, the proportion of Rami + microglia is greater in M1 than in PMC of non-lesion control (Fig. 8c, Fisher’s LSD post hoc, ctr. %Rami + M1 vs. PMC: *p < *0.01). Lesion in the vehicle group shifted this distribution of microglial subtypes to the opposite pattern, with %Rami + microglia lower in M1 than in PMC (Fig. 8c, Fisher’s LSD post hoc, %Rami + veh., M1 vs. PMC: *p < *0.05). Lesion in the vehicle group also showed a relative increase in %Hyper- microglia in M1 compared to PMC, that did not differ significantly in the non-lesion control brains (Fig. 8c, Fisher’s LSD post hoc, M1 vs. PMC %Hyper- ctr: *p = *0.07; veh: *p < *0.05). Interestingly, EV treatment seemed to dampen this lesion-related decrease in %Rami + and increase in % Hyper- microglia in M1 relative to PMC (Fig. 8c).

In summary, between-group, within-area, comparisons showed that in M1, lesion proportionally decreased ramified microglia, but increased hypertrophic microglia compared to non-lesion control (Figs. 7 and 8). The opposite pattern was seen in PMC, where lesion shifted to a proportional increase in C1q + ramified microglia but a decrease in C1q- hypertrophic microglia (Fig. 8a–c). Further, there are between-area differences in the normative distribution of these microglia. Our results also demonstrated that EV treatment regulated the post-injury region-dependent expression of microglia subtypes by reversing or attenuating the lesion associated changes.

### Microglia morphological features are dependent on experimental group and C1q expression

Overall, our results indicated that lesion and EV treatment facilitated a specific phenotypic shift in gray matter C1q + vs C1q- hypertrophic, and to a lesser extent ramified, microglia in a region dependent manner. While we had classified microglia as distinct categorical types based on a combination of somewhat qualitative features described in the literature [39], microglia morphology is a continuum. The transition states between these microglial classes and the quantitative morphological differences remain unclear. Thus, we further assessed and validated whether our user-classified C1q + vs C1q- hypertrophic and ramified microglia indeed represented distinct subclasses based on quantitative morphologic features. We quantified a subset of morphological features (soma size, aspect ratio, and number and orientation of primary processes) of individual microglia in M1 partially reconstructed in 3D in 60-µm sections. Using a three-way ANOVA, we assessed the independent and interactive effects of 'morphology*C1q expression*experimental group' (Fig. 8d–g). We were not able to report reconstruction data for C1q- hypertrophic microglia in control monkeys since this subpopulation was very rare in this group. There were significant main effects of 'group' and 'morphology' on the number of primary processes (Fig. 8e: three-way ANOVA, ‘group’/‘morphology’ main effect, *p < *0.05; Table S3 reports exact p-values for significant comparisons). Specifically, microglia in both groups with lesion had a greater number of primary processes as compared to the controls. Ramified C1q + microglia in both groups with lesion showed a greater number of primary processes as compared to the controls (Fig. 8e, Fisher’s LSD post hoc, Rami + : ctr. vs. veh and ctr. vs. EV: *p < *0.05). In addition, there was a treatment effect on hypertrophic C1q ± microglia, which exhibited a greater number of processes in the EV-treated group compared to those in vehicle and control groups (Fig. 8e, Hyper + : ctr. vs. EV and veh. vs. EV: *p < *0.05; Hyper-: veh. vs. EV: *p < *0.05). For all C1q ± microglia in the EV group, the hypertrophic microglia had greater numbers of primary processes as compared to the ramified cells (Fig. 8e, Hyper + vs. Rami + and Hyper- vs. Rami-: *p < *0.05). These results demonstrated that regardless of C1q expression, microglia from the EV-treated group was characterized by a greater number of primary processes, consistent with our previous work [13].

Significant main effects of 'C1q expression ( ±)' and 'group' were found for features of ramified and hypertrophic microglia somata (Fig. 8f: three-way ANOVA, ‘group’/‘C1q expression’ main effect, *p < *0.01). Both hypertrophic and ramified C1q + microglia in EV group but not the veh group had greater surface area of their soma as compared to the non-lesion control group (Fig. 8f, Fisher’s LSD post hoc, Hyper + and Rami + ctr. vs. EV *p < *0.05). In addition, a significant main effect of C1q expression was found for cell body aspect ratio, which was calculated as the maximum diameter of the microglial somata divided by the minimum diameter (Fig. 8g, three-way ANOVA, main effect, ‘C1q expression’, *p < *0.05). Aspect ratio can be a measure of microglia polarization and immune activation [48]. Cell bodies of C1q + microglia (Rami + or Hyper +) exhibited a smaller aspect ratio (rounder) compared to the C1q- microglia which had a more elongated, oval shape somata. These results showed that C1q expression on microglia was associated with rounder somata with larger surface areas.

We then assessed whether these unique sets of morphological features will reveal distinct clusters of microglia subtypes. For each microglia reconstructed, we plotted a 3D scatter plot of primary process, soma volume, and soma aspect ratio, and annotated the plots based on morphology and C1q expression, cortical region and experimental group (Fig. 8h). 3D scatter plot revealed some clustering of microglia based mainly on morphology and not C1q expression (Fig. 8h). Specifically, ramified microglia were associated with smaller soma volume and rounder somata shape (smaller aspect ratio) as compared to the hypertrophic microglia (Fig. 8h, left panel). Our clustering analysis also showed that microglia in the three groups exhibited different morphologies (Fig. 8h, right panel). The non-lesion controls tend to have smaller soma with a broader range of aspect ratio and less primary processes. Microglia in the EV group was shown to have larger soma and more processes, whereas the vehicle group had intermediate soma volume but a higher aspect ratio indicating an oval somata shape (Fig. 8h right panel). In contrast to morphological categories and experimental group, no clustering of microglia based on these three morphological features was found between regions (Fig. 8h, middle panel).

**Fig. 9:**
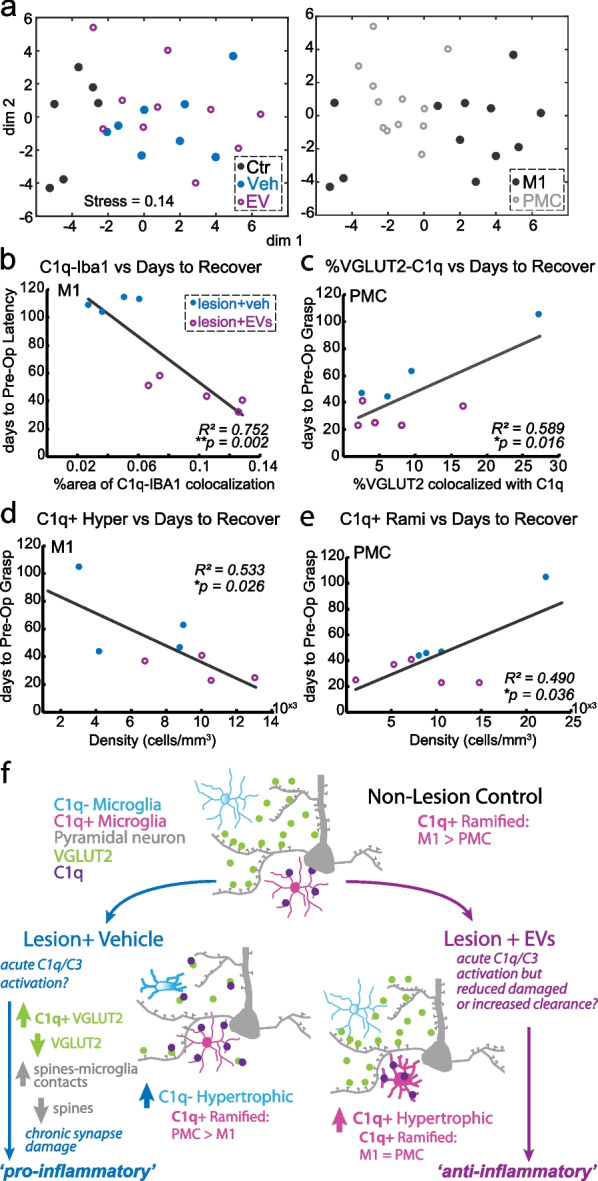
Relationship of synaptic and microglia properties to behavior outcome measures. **a** NMDS plots showing clustering of cases, annotated by experimental group (left) and cortical area (right), based on 21 synaptic and microglia outcome measures (%area VGLUT1, VGLUT2, VGAT, GLUR2/3, GABAA alpha1, GABAB R2; % of VGLUT1, VGLUT2 or VGAT with Iba1; % of Iba1 with VGLUT1, VGLUT2 or VGAT; % area C1q; % of VGLUT2 with C1q; % C1q with VGLUT2; cell densities of ramified, hypertrophic, amoeboid C1q + and C1q- microglia). The proximity of points indicates the relative similarity-based on pair-wise correlation of these multiple variables. **b-e** Significant linear correlations between synaptic–microglial measures and behavioral outcome measures: **b** increased density of C1q and Iba1 colocalization in M1 correlated with faster recovery time (less days to return to pre-operative latency to retrieve food reward; *R*^*2*^ = 0.752, *p = *0.002). **c** Increased fraction of VGLUT2 colocalized with C1q in PMC correlated with slower recovery time (more days return to preoperative grasp pattern; *R*^*2*^ = 0.589, *p = *0.016). **d** Greater expression of C1q + hypertrophic microglia in M1 was correlated with faster recovery time (*R*^*2*^ = 0.533, *p = *0.026).** d** Greater expression of C1q + ramified microglia in PMC was associated with slower recovery time (*R*^*2*^ = 0.490, *p = *0.036). **f** A schematic showing summary of findings and proposed model of the lesion and EV treatment effects on microglia–synapse modulation and C1q signaling pathways. Cortical lesion in M1 induces acute damage in neuronal structures that triggers an acute increase in C1q + signaling cascade to initiate phagocytotic clearance. The veh group had accumulation of further damage and downstream C1q pathway related proteins that sustains a chronic pro-inflammatory response (C1q- hypertrophic microglia). The EV treatment upregulated C1q + mediated clearance of debris and facilitated an early shift to the anti-inflammatory C1q + hypertrophic microglia phenotype that persisted in the chronic stages, thereby supporting functional recovery

We then determined the combined effects of synaptic and microglia outcome measures on the relative (dis)similarities of areas and experimental groups in this study. Thus, we performed non-metric multidimensional scaling and clustering of individual PMC (average of two fields per case) and M1 (average of four fields per case) imaging fields from each case (*n = *3 Ctr, 4 veh, 5 EV), based on a distance proximity matrix derived from pair-wise correlation of 21 synaptic and microglia outcome measures (Fig. 9a: per case: % area VGLUT1, VGLUT2, VGAT, GLUR2/3, GABA_a_ α1, GABA_b_ R2; % of VGLUT1, VGLUT2 or VGAT with Iba1; % of Iba1 with VGLUT1, VGLUT2 or VGAT; % area C1q; % of VGLUT2 with C1q; % C1q with VGLUT2; cell densities of ramified, hypertrophic, amoeboid C1q + and C1q- microglia). Our analyses note that there is a strong separation between the control from the lesion group (Fig. 9a). Further, within each experimental group, M1 vs PMC are separated (Fig. 9a). This regional separation is most prominent within the non-lesion control group, highlighting the normative diversity between cortical areas with regard to synaptic and microglial features [34, 49].

### Microglial C1q expression and C1q-VGLUT2 tagging correlated with functional recovery after cortical injury

Our previous study [12] has reported that compared with vehicle-treated monkeys, EV-treated monkeys exhibited enhanced recovery of fine motor function of the hand, evidenced by the fewer number of days to return to pre-operative hand grasp pattern and latency to retrieve food reward. Using the functional recovery data from Moore et al. and Pessina et al. [12, 40], we used linear regression analyses to determine whether the cellular data reported above were associated with behavioral measures of motor recovery (number of days to return to pre-operative latency and grasp pattern). Our results showed that increased C1q-Iba1 colocalization in M1—specifically increased density of C1q + hypertrophic microglia—was associated with a more rapid recovery rate (fewer days to return to pre-operative latency; Fig. 9b, R^*2*^ = 0.752, *p < *0.01; Fig. 9d, R^*2*^ = 0.533, *p < *0.05). In PMC, we found increased portion of VGLUT2 tagged by C1q in PMC was associated with a slower recovery rate (more days to return to preoperative grasp pattern; Fig. 9c, R^*2*^ = 0.589, *p < *0.05). Increased density of C1q + ramified microglia in PMC was also associated with a slower recovery rate (more days to return to preoperative grasp pattern, Fig. 9e, R^*2*^ = 0.49, *p < *0.05). Overall, our results indicated that the C1q + hypertrophic microglial expression in perilesional M1 was beneficial for the recovery of fine motor function. In contrast, C1q tagging on VGLUT2 + synapse in PMC was detrimental for recovery, indicated by the slower recovery rate.

## Discussion

Previous studies have demonstrated the effects of EVs on shifting microglial morphological phenotypes [13] and ameliorating synaptic imbalance in cortical and spinal motor circuits [14, 50], to support recovery of motor function after cortical injury in the primary motor cortex (M1). The current study provides a mechanistic link between these previous data, showing complementary effects of EV treatment on synaptic marker and microglial expression, and their structural and molecular relationships. As summarized in Fig. 9f, our data suggest that the surgical lesion and EV treatment post-injury each affect microglia–synapse relationships and microglial phenotypic expression of the complement pathway initiator protein, C1q, in a region-dependent manner. Compared to vehicle, EV treatment was associated with increased expression of C1q + hypertrophic microglia and decreased expression of C1q- hypertrophic microglia in M1, but decreased expression of C1q + ramified microglia and C1q synapse tagging in PMC (Fig. 9f). These data point to the role of EV-mediated regulation of region and circuit-specific synaptic plasticity to support recovery of motor function after cortical injury.

### Differential effects of injury on excitatory VGLUT1 and VGLUT2 terminals suggest pathway-specific mechanisms for plasticity

Cortical injury leads to neuronal hyperexcitability, excitotoxicity, and disruption of synaptic transmission in perilesional cortex [3, 14]. Glutamate, the major excitatory neurotransmitter in the central nervous system, is stored and transported into synaptic vesicles by presynaptic vesicular glutamate transporters (VGLUTs) that have isoforms differentially expressed across distinct neuronal pathways [51]. In the adult brain, VGLUT1 is expressed mainly by cortico-cortical axons, while VGLUT2 is mainly expressed in subcortical glutamatergic neurons, predominantly in thalamocortical axons [52]. The current results showed significant reduction of VGLUT2 in both groups with lesions compared to non-lesion controls, while no significant difference was found in VGLUT1. These results suggest that either axon terminals expressing VGLUT2 + may be selectively vulnerable to injury compared to VGLUT1 + , or that VGLUT1 + connections have a greater degree of plasticity after injury. This is consistent with literature suggesting that axon terminals expressing VGLUT1 such as those in the hippocampus exhibit higher potential for plasticity than those expressing VGLUT2, such as the climbing fibers in the cerebellum [53]. Future experiments will be important to clarify the molecular pathways underlying the differential regulation of VGLUT1 and VGLUT2 expression after injury.

### EV treatment ameliorated injury-related dysregulation of glutamate AMPA receptor subunit gene expression in PMC

Glutamatergic AMPA receptor composition is an important determinant of synaptic strength and plasticity [3]. The GLUR2 AMPAR subunit, in particular, controls calcium permeability, thereby affecting AMPARs trafficking, and spine growth [54, 55]. In the present study, lesion-related reduction of GLUR2/3 receptors is dampened in the EV-treated group. However, mRNA expression of *GRIA2,* the gene for GLUR2 AMPA receptor subunit, showed the opposite trend; the lesion-related increase in *GRIA2* mRNA was downregulated and normalized by EV. The opposite effects of lesion on GLUR2/3 protein and *GRIA2* mRNA may be due to numerous factors. First, the *GRIA2* mRNA is related to GLUR2 protein translation, but not GLUR3 receptors. Second, the mRNA–protein discrepancy suggests differences in transcriptional and post-translational regulation of plasticity in response to lesion and lesion-induced hyperexcitability. Previous work in rodents has shown that increased protein expression of GLUR2 promotes dendritic spines formation and enlargement in rat hippocampal neurons [56]. Upregulation of *GRIA2* mRNA and concomitant downregulation of *GRIA1* (GLUR1) mRNA was found in response to pharmacologically induced hyperexcitability in neuronal cultures [57]. The current data are consistent with our previous findings showing lesion-induced hyperexcitability, accompanied by excitatory synapse loss at the electrophysiological and structural level in ventral PMC [14]. Thus, it is possible that lesion-related *GRIA2* mRNA upregulation resulted from increased hyperexcitability, but the downstream mechanisms for GLUR2/3 subunit protein synthesis, trafficking and insertion were impaired, thereby not allowing for spine and synapse growth after injury. Interestingly, the current data suggest that this potential impairment in post-translational regulation is apparent only in vehicle monkeys, where upregulated GLUR2 mRNA was found together with downregulated GLUR2/3 protein expression. In EV-treated monkeys, GLUR2 mRNA and GLUR2/3 protein levels, which showed the opposite trend from vehicle monkeys, were normalized closer to baseline non-lesion control. This is consistent with our previous work demonstrating that the lesion-related decrease in excitatory postsynaptic current frequencies and spine loss were ameliorated by EVs [14]. Together, these data suggest that EV-mediated dampening of chronic hyperexcitability in perilesional neurons, can be associated with preventing aberrant plasticity and maintaining glutamatergic synapse growth.

### Differential effects of injury on distinct inhibitory neurotransmitter receptor expression

In addition to changes in excitatory neurotransmission, phasic and tonic inhibitory GABAergic transmission, conferred by distinct ionotropic GABA_a_ and metabotropic GABA_b_ receptor subunits [58], also play a complex role in recovery after injury [59, 60]. Here, we found in both M1 and PMC a lesion-related reduction in the density of GABA_a_ α1, a subunit localized on synaptic membranes mediating fast phasic inhibitory currents [58]. Further, in our qPCR results, we found a lesion-related increase in gene expression of GABA_a_ ∂ (*GABRD*) and GABA_b_ R2 (*GABBR2*) subunits, known to mediate tonic inhibitory currents that control overall cell excitability [3, 43, 61]. The current results are consistent with previous studies in rodent and in vitro models showing that injury results in increased tonic inhibition to prevent excitotoxicity during the acute recovery period [59, 60]. However, re-establishing phasic inhibitory transmission is needed to support reorganization [59, 60]. While the current data did not show a treatment effect with regard to GABA receptors, our previous study showed that EV treatment was associated with a specific increase in distal apical inhibitory synapses and task-related immediate early gene activation of dendritic-targeting inhibitory interneurons that support recovery of motor function [14]. Thus, cell-type and compartment specific changes in GABAergic receptor expression across recovery are important to assess in future work.

### EV treatment upregulated the anti-inflammatory C1q + hypertrophic microglia in M1

While activity-dependent, neuronal mechanisms of synaptic plasticity have been well studied, it is only recently that the role of microglia and neuro-immune signaling have been investigated [62, 63]. In the healthy brain, microglia can regulate synaptic turn-over through phagocytosis—likely partial (i.e., trogocytosis [27])—of synapses for pruning, or releasing trophic factors to promote synapse growth [62, 64]. After injury, microglia, as the resident macrophages of the brain, are stimulated to mediate clearance of damaged synapses [65–68]. A crucial part of this microglial mediated neuro-immune signaling is the complement system [26, 37]. During periods of active synaptic pruning or after injury, the initiating protein of the classical complement cascade, C1q, is produced by microglia to tag excess or damaged synapses. C1q tagging then triggers downstream deposition and activation of complement effector molecules, such as C3 receptors, which would in turn lead to microglial synapse phagocytosis [46, 47, 69]. In the present study, the vehicle group showed a lesion-related elevation in C1q tagging of VGLUT2 + axon terminals (C1q + /VGLUT2 + colocalization), coupled with decreased VGLUT2 + density, suggesting greater synapse damage and loss [14]. However, in the EV group, this C1q-synapse tagging was dampened, and coupled with a greater expression of C1q + hypertrophic microglia in perilesional M1 (Fig. 9f). After cortical injury, microglia can be ‘immune-activated’, partially through the complement pathway, to exhibit distinct macrophage-like phenotypes polarized towards either pro- and anti-inflammatory functions [30–32, 70]. Synthesis of C1q within anti-inflammatory (M2 macrophage-like) microglia has been shown to be acutely upregulated after cortical injury, playing a protective role, to promote the clearance of apoptotic cells and secretion of anti-inflammatory cytokines, and suppress production of pro-inflammatory cytokines [6, 8, 28, 29]]. Thus, EV treatment mitigated a sustained chronic pro-inflammatory state, evidenced by increased expression of the protective and debris-clearing C1q + hypertrophic microglia in perilesional cortex, 12 weeks post-injury. Indeed, here we show that greater expression of C1q + hypertrophic microglia in perilesional M1 was associated with more rapid recovery of function. Interestingly, we found that the vehicle group exhibited a decrease in the proportion of C1q + but an increase in C1q- hypertrophic microglia in perilesional M1, compared to EV and control groups (Fig. 9f). Thus, these C1q- hypertrophic microglia represents a distinct population, likely belonging to the pro-inflammatory subclass [30, 31], which persisted in the vehicle monkeys. These pro-inflammatory microglia can exacerbate neurotoxicity by releasing pro-inflammatory cytokines including TNF-α, IL-6, and IL-1β [4, 71].

In a mouse model of Alzheimer’s disease [72], it was found that early in the disease, C1q ‘primed’ microglia are anti-inflammatory and protective. However, after C1q release and tagging, the downstream molecular targets of C1q can either promote anti-inflammatory (via C3b receptor activation) or exacerbate chronic pro-inflammatory (via C3a or C5 receptor activation) signaling [47]. Future studies to assess the temporal progression of distinct C1q effectors will be important to further understand the role of EVs in modulating the complement system to support recovery after cortical injury. Nevertheless, the current findings together with our previous data [13] support the role of EV treatment in inducing an early shift from pro-inflammatory to anti-inflammatory microglia in perilesional cortex, preventing chronic inflammation and damage after cortical injury.

### Region-dependent effects of injury and treatment in modulating microglial phagocytosis

The current findings revealed novel region-specific expression of microglial phenotypes that are differentially modulated by injury and EV treatment. In the non-injured brain, ramified homeostatic microglia predominated. However, there were baseline regional differences in the proportion of C1q + vs C1q- ramified microglia, with M1 showing a significantly greater proportion of C1q + microglia than PMC. These data are consistent with previous work in the rodent and human brain, highlighting diversity across cortical areas with regard to microglial subpopulations [73–76]. Further, the present findings suggest innate differences in microglial/C1q dependent synapse turnover between two cortical motor areas, with synapses in M1 likely subject to greater synaptic pruning via the upregulated C1q + ramified microglia compared to PMC [63, 77]. Interestingly, the lesion alone resulted in a reversal of the M1 vs PMC gradient in C1q + ramified microglia; However, EV treatment mitigated this lesion-related regional shift. Indeed, while increasing expression of C1q + hypertrophic microglia in M1 was associated with a more rapid recovery rate, increased C1q-synapse (VGLUT2) tagging and C1q + ramified microglia in PMC were associated with a prolonged recovery. These results suggest that microglia phagocytosis can be beneficial in perilesional M1, which likely reflect the neuroprotective effects of clearance of damaged tissue necessary to promote tissue repair and re-establish homeostasis after injury [4]. However, chronic and excessive phagocytosis of synapses in PMC can exacerbate neuronal cell death and loss of connections [5, 62, 63, 78] that can be detrimental to recovery.

Previous data have shown that axon terminals can be engulfed and phagocytosed by microglia [64]. However, microglia closely apposed or proximal to synaptic elements can alternatively indicate microglia–synapse signaling to release neurotrophic factors [4] or promote trogocytosis, which is a process of partial phagocytosis of synapses via microglial processes [27]. It is possible that the enhancement of C1q + hypertrophic microglia with EV treatment promote these non-canonical mechanisms of microglial-mediated synapse pruning and plasticity that do not involve classical phagocytosis, which can be addressed in future work.

### Limitations and future directions

While the current study has described microglia–synapse overlap and profiled microglia subclasses in distinct cortical areas using combined quantitative and proteomic expression criteria, we recognize that our approach is still limited. First, in our microglia profiling, it is important to note that we have only measured a few quantitative 3D morphological features (due to using relatively thin sections) and we have only looked at expression of two markers (Iba1 and C1q) in two cortical regions. Future work to expand on detailed morphological and proteomic profiling of microglia across diverse brain regions in the primate brain will be important to build our understanding of primate-specific cell diversity as it relates to neuro-immune responses.

Second, a major limitation of the current study is the use of a cohort of female lesion monkeys, and unavailability of male monkeys for lesion studies at the time of experimentation and tissue collection. Indeed, while we have not found sex differences in age-related changes in our previous work [79], we have found significant sex differences in the extent and nature of recovery after cortical injury in male versus female aged monkeys, using metanalyses of multiple archived datasets from our group [80]. Since sex differences in microglia profiles have been observed in previous work [81–83], future studies assessing the effects of sex on microglia and synapse outcome measures are underway. Further, it is also notable that the lesion monkeys used in this study are considered within late middle-aged to aged. On the one hand, this shows the surprising plasticity and resilience of cortex to damage even in late middle age, an age that has a high occurrence of cortical injury in humans [84]. On the other hand, it remains unknown how the levels and forms of plasticity identified here differ from young adults or from older monkeys over 25 years of age, when very severe cognitive deficits are apparent [85].

Finally, the current study has some limitations with regard to the nature of the visualization of microglial–synapse interaction and sampling volume. While the structural overlap of microglia and synaptic labeling studied here at the confocal level can be indicative of microglial–synapse interactions, it is important to note that this method is static and is limited in resolution. Thus, future studies can employ live-cell imaging methods to visualize dynamic processes of microglial activity, and super resolution or electron microscopy techniques to validate subcellular interactions, including the occurrence of phagocytosis [86–88]. On the other hand, it is also important to note that the use of high-resolution imaging at the limits of diffraction [89] to visualize and quantify subcellular structures in the current study inherently limits sampling volume. Further, due to the limited availability of tissue sections, some markers were only quantified in one section, albeit across multiple fields. For such imaging methods, sampling at high resolution for precise visualization of small particles is inadvertently prioritized over stereological 3D whole-brain sampling using multiple serial sections; This is in line with 3D counting principles (i.e., size-frequency) postulating that quantifying particles much smaller in size than the section thickness or sample volume yields reliable unbiased estimates of density [90, 91]. Nevertheless, it is important to recognize this limitation and consider consistent volumetric sampling to account for regional differences, as we have done in the current study. Indeed, our data show intrinsic between-region (PMC vs M1) differences in synaptic and neuro-immune markers, highlighting the importance of future work on the regional specificity of responses to injury and mechanisms for plasticity in the primate brain [77].

## Conclusion

We have elucidated the potential microglia–synapse and complement C1q-related mechanisms of how MSC-EVs treatment can enhance recovery in a monkey model of cortical injury. Cortical lesion results in reduced expression of excitatory and inhibitory synaptic markers in perilesional M1 and PMC, consistent with the functional deficits in excitatory and inhibitory synaptic transmission shown in monkeys [14] and rodents [59]. The current data suggest that EV treatment facilitated synaptic plasticity by regulating microglial activity in a region-dependent manner—enhancing anti-inflammatory C1q + hypertrophic microglia expression in perilesional M1 for clearance of acute damage, and thereby preventing excessive synaptic loss in PMC and chronic synaptic dysfunction. These mechanisms may act to preserve synaptic cortical motor networks and balanced normative M1/PMC synaptic connectivity to support functional recovery after injury [14, 23, 92, 93]. However, it still remains unclear whether EVs act on microglia directly or indirectly via upstream signaling pathways [4] or via suppressing pro-inflammatory complementary system markers downstream to C1q. Our findings in this current study form an important basis for future work to dissect the molecular effects of MSC-EVs treatment on the complement cascade crucial for recovery after cortical injury, and extend their therapeutic application for acute and chronic neurodegenerative diseases.

### Supplementary Information

**Additional file 1: Figure S1.** Representative maximum-projection confocal images (with 5-8 optical stacks) of negative (Neg) and positive controls of immuno-markers in dorsal PMC (negative controls) and sublesional M1 (with immuno-staining) of one animal from each experimental group (*n = *1 ctr, 1 veh, 1 EV-treated animal). a-VGLUT1, b-VGLUT2, c-GLUR2/3, d-VGAT, e- GABA_a_ a1, f- GABA_b_ R2, g-C1q, h-Iba1 & P2RY12), scanned with a 40 × lens. Scale bar: 20 µm. **Table S1.** Number of subjects and marker combinations used for IHC batches. **Table S2.** Number of sections from each subject used for each immuno-stained marker. **Table S3. **Summary of p-values showing significant ANOVA main effects, interactions and post hoc tests for microglial morphological parameters in Fig. 8.

## Data Availability

The raw data that support the findings in the present study are available from the corresponding author upon request.

## Abbreviations

A-/Ame-: Amoeboid microglia without C1q expression
A + /Ame +: Amoeboid microglia with C1q expression
AMPA: α-Amino-3-hydroxy-5-methyl-4-isoxazolepropionic acid
BSA: Bovine serum albumin
Ctr: Non-lesion control
EVs: Extracellular vesicles
GABA: γ-Aminobutyric acid
GLUR: Glutamate receptor
H-/Hyper-: Hypertrophic microglia without C1q expression
H + /Hyper +: Hypertrophic microglia with C1q expression
Iba1: Ionized calcium binding adaptor molecule 1
IHC: Immunohistochemistry
L3: Layer 3
M1: Primary motor cortex
MG: Microglia
MSC-EVs: Mesenchymal stem cell-derived extracellular vesicles
NDS: Normal donkey serum
NMDS: Nonmetric multidimensional scaling
P2RY12: Purinergic receptor P2Y, G-protein coupled, 12
PB: Phosphate buffer
PFA: Paraformaldehyde
PMC: Premotor cortex
R-/Rami-: Ramified microglia without C1q expression
R + /Rami +: Ramified microglia with C1q expression
Veh: Vehicle group
VGAT: Vesicular GABAergic transporters
VGLUT: Vesicular glutamate transporter
