# Supplementary material for: Mesenchymal-derived extracellular vesicles enhance microglia-mediated synapse remodeling after cortical injury in aging Rhesus monkeys
Source: J Neuroinflammation. 2023 Sep 2;20:201. doi: 10.1186/s12974-023-02880-0 (PMC10475204; doi:10.1186/s12974-023-02880-0)
Supplement: Supplementary file 1 — Additional file 1: Figure S1. Representative maximum-projection confocal images (with 5-8 optical stacks) of negative (Neg) and positive controls of immuno-markers in dorsal PMC (negative controls) and sublesional M1 (with immuno-staining) of one animal from each experimental group (n = 1 ctr, 1 veh, 1 EV-treated animal). a-VGLUT1, b-VGLUT2, c-GLUR2/3, d-VGAT, e- GABAa a1, f- GABAb R2, g-C1q, h-Iba1 & P2RY12), scanned with a 40 × lens. Scale bar: 20 µm. Table S1. Number of subjects and marker combinations used for IHC batches. Table S2. Number of sections from each subject used for each immuno-stained marker. Table S3. Summary of p-values showing significant ANOVA main effects, interactions and post hoc tests for microglial morphological parameters in Fig. 8. [file 12974_2023_2880_MOESM1_ESM.docx]

**
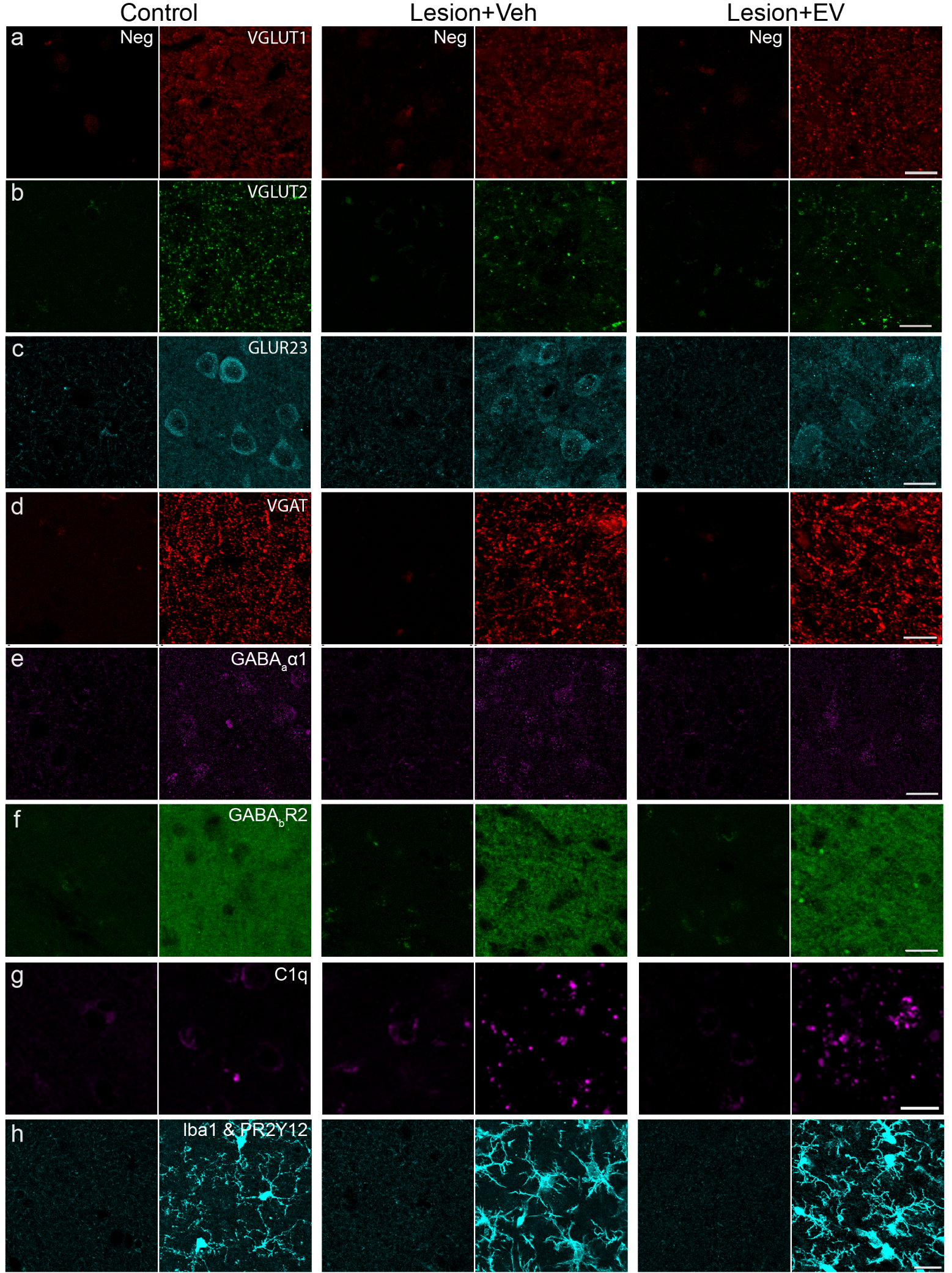
**

**Figure S1:** Representative maximum-projection confocal images (with 5-8 optical stacks) of negative (Neg) and positive controls of immuno-markers in dorsal PMC (negative controls) and sublesional M1 (with immuno-staining) of one animal from each experimental group (n= 1 ctr, 1 veh, 1 EV-treated animal). a-VGLUT1, b-VGLUT2, c-GLUR2/3, d-VGAT, e- GABA_a_ a1, f- GABA_b_ R2, g-C1q, h-Iba1 & P2RY12), scanned with a 40x lens. Scale bar: 20 µm

**Table S1: Number of subjects and marker combinations used for IHC batches.**

| IHC Batch | Animals (n) | Channel 1 | Channel 2 | Channel 3 | Channel 4 |
| --- | --- | --- | --- | --- | --- |
| 1 | ctr=3, veh = 5, EV=5 | **VGAT (guinea pig)** | **GABA_a_ a1(rabbit)** | **GABA_b_ R2 (mouse)** | Parvalbumin (goat) |
| 2 | ctr=3, veh = 4, EV=5 | **VGLUT1 (goat)** | **VGLUT2 (guinea pig)** | **Iba1/P2RY12 (rabbit)** | N/A |
| 3 | ctr=3, veh = 4, EV=5 | **VGLUT1 (goat)** | **VGLUT2 (guinea pig)** | **Iba1 (rabbit)** | **C1q (mouse)** |
| 4 | ctr=3, veh = 4, EV=5 | **VGAT (guinea pig)** | **Iba1/P2RY12 (rabbit)** | **C1q (mouse)** | N/A |
| 5 | ctr=3, veh = 4, EV=5 | **GLUR2/3 (rabbit)** | GLUR1 (mouse) | Cfos (guinea pig) | Parvalbumin (goat) |
| 6 | ctr=3, veh = 4, EV=5 | **GLUR2/3 (rabbit)** | SMI (mouse) | Cfos (guinea pig) | Parvalbumin (goat) |

Table S1 summarizes the combination of markers labeled for each of the 6 batches of IHC experiments and the number of subjects per experimental group. Markers in bold are reported in the present study

**Table S2:** **Number of sections from each subject used for each immuno-stained marker.**

| **Group** | **ID** | **Age** | **Sex** | **number of sections per animal and marker** | | | | | | | | |
| --- | --- | --- | --- | --- | --- | --- | --- | --- | --- | --- | --- | --- |
|  |  |  |  | **VGLUT1** | **VGLUT2** | **GLUR2/3** | **VGAT** | **GABA_a_ α1** | **GABA_b_ R2** | **P2RY12** | **Iba1** | **C1q** |
| Non-lesion Ctr | AM311 | 20.3 | M | 2 | 2 | 2 | 2 | 1 | 1 | 2 | 3 | 2 |
|  | AM342 | 19.2 | F | 2 | 2 | 2 | 2 | 1 | 1 | 2 | 3 | 2 |
|  | AM350 | 17.3 | F | 2 | 2 | 2 | 2 | 1 | 1 | 2 | 3 | 2 |
| Lesion-Veh | AM323 | 24 | F | 2 | 2 | 2 | 2 | 1 | 1 | 2 | 3 | 2 |
|  | AM331 | 26.5 | F | 2 | 2 | 2 | 2 | 1 | 1 | 2 | 3 | 2 |
|  | AM335 | 20.8 | F | 2 | 2 | 2 | 2 | 1 | 1 | 2 | 3 | 2 |
|  | AM337 | 24.8 | F | - | - | - | 1 | 1 | 1 | - | - | - |
|  | AM339 | 21.9 | F | 2 | 2 | 2 | 2 | 1 | 1 | 2 | 3 | 2 |
| Lesion-EV | AM320 | 22.5 | F | 2 | 2 | 2 | 2 | 1 | 1 | 2 | 3 | 2 |
|  | AM332 | 24.5 | F | 2 | 2 | 2 | 2 | 1 | 1 | 2 | 3 | 2 |
|  | AM338 | 16.9 | F | 2 | 2 | 2 | 2 | 1 | 1 | 2 | 3 | 2 |
|  | SM061 | 22.1 | F | 2 | 2 | 2 | 2 | 1 | 1 | 2 | 3 | 2 |
|  | SM062 | 21.3 | F | 2 | 2 | 2 | 2 | 1 | 1 | 2 | 3 | 2 |

Table S2 summarizes the total number of sections through the lesion used for each marker labeled, reported with their animal ID, experimental group, age, and sex

**Table S3: Summary of p-values showing significant ANOVA main effects, interactions and post-hoc tests for microglial morphological parameters in Figure 8.**

| **Outcome measure** | **Types of statistical analysis** | **Comparison** | **p-values** |
| --- | --- | --- | --- |
| %Rami+ microglia out of all microglia | Two-way ANOVA (group*area)  Fisher’s LSD *post hoc* | ***Interaction*** | **‘Group*Area’: p=0.008** |
|  |  | *Between-area* within Ctr/Veh | **Ctr: M1 > PMC: p=0.009**  **Veh: PMC > M1: p=0.05** |
|  |  | *Between-group* within M1 | **M1: Ctr > Veh: p=0.006**  **Ctr > EV: p=0.004** |
| %Hyper+ microglia out of all microglia | Two-way ANOVA  (group*area)  Fisher’s LSD *post hoc* | ***Main effect*** | **‘Group’: p=0.01** |
|  |  | *Between-group* within M1 | **EV > Ctr: p=0.01** |
| %Hyper- microglia out of all microglia | Two-way ANOVA  (group*area)  Fisher’s LSD *post hoc* | ***Interaction*** | **‘Group*Area’: p=0.015** |
|  |  | *Between-area* within Veh | **Veh: M1 > PMC: p=0.013** |
|  |  | *Between-group* within M1 | **M1: Veh > Ctr: p=0.006** |
| %Total C1q+ microglia | Two-way ANOVA  (group*area)  Fisher’s LSD *post hoc* | ***Interaction*** | **‘Group*Area’: p=0.04** |
|  |  | *Between-area* within Ctr | **Ctr: M1 > PMC: p= 0.02** |
|  |  | *Between-group* within PMC | **PMC: Veh > Ctr: p=0.02** |
| %Hyper+ microglia out of all Hyper microglia | Two-way ANOVA  (group*area)  Fisher’s LSD *post hoc* | ***Interaction*** | **‘Group*Area’: p=0.004** |
|  |  | *Between-group* within M1 | **M1: Ctr > Veh: p=0.001**  **Ctr > EV: p=0.03**  **EV> Veh: p=0.006** |
| Number of primary processes (microglia in M1) | Three-way ANOVA  (group*morpho*C1q)  Fisher’s LSD *post hoc* | ***Main effect*** | **‘Group’: p=0.01** |
|  |  | *Between-group* within microglia morphology/ C1q | **Hyper/C1q+: EV > Ctr: p=0.03; EV > Veh: p=0.05**  **Hyper/C1q-: EV > Veh: p=0.02**  **Rami/C1q+: Veh > Ctr: p=; EV > Ctr: p=0.03** |
|  | Three-way ANOVA  (group*morpho*C1q)  Fisher’s LSD *post hoc* | ***Main effect*** | **‘Morphology’: p=0.02** |
|  |  | *Between-morphology type* within group/C1q | **EV/C1q+: Hyper+ > Rami+: p=0.004**  **EV/C1q-: Hyper- > Rami -: p<0.001** |
| Soma surface area  (microglia in M1) | Three-way ANOVA  (group*morpho*C1q)  Fisher’s LSD *post hoc* | ***Main effects*** | **‘C1q expression’: p=0.004**  **‘Group’: p=0.006** |
|  |  | *Between-group* within C1q | **Hyper/C1q+: EV > Ctr: p=0.01**  **Rami/C1q: EV > Ctr: p=0.04** |
| Aspect ratio  (microglia in M1) | Three-way ANOVA  (group*morpho*C1q)  Fisher’s LSD *post hoc* | ***Main effect*** | **‘C1q expression’: p=0.02** |
|  |  | *Between-*C1q within group/morphology | **EV/Rami: C1q+ > C1q-, p=0.026** |

Table S3 summarizes the p-values for significant differences or approaching significance, from Two-way and Three-way ANOVA and *post-hoc* comparisons of microglial parameters in Figure 8
